# Constructing robust and efficient experimental designs in groundwater modeling using a Galerkin method, proper orthogonal decomposition, and metaheuristic algorithms

**DOI:** 10.1371/journal.pone.0254620

**Published:** 2021-08-05

**Authors:** Timothy T. Ushijima, William W. G. Yeh, Weng Kee Wong

**Affiliations:** 1 Department of Civil and Environmental Engineering, University of California, Los Angeles, California, United States of America; 2 Department of Biostatistics, University of California, Los Angeles, California, United States of America; Torrens University Australia, AUSTRALIA

## Abstract

Estimating parameters accurately in groundwater models for aquifers is challenging because the models are non-explicit solutions of complex partial differential equations. Modern research methods, such as Monte Carlo methods and metaheuristic algorithms, for searching an efficient design to estimate model parameters require hundreds, if not thousands of model calls, making the computational cost prohibitive. One method to circumvent the problem and gain valuable insight on the behavior of groundwater is to first apply a Galerkin method and convert the system of partial differential equations governing the flow to a discrete problem and then use a Proper Orthogonal Decomposition to project the high-dimensional model space of the original groundwater model to create a reduced groundwater model with much lower dimensions. The reduced model can be solved several orders of magnitude faster than the full model and able to provide an accurate estimate of the full model. The task is still challenging because the optimization problem is non-convex, non-differentiable and there are continuous variables and integer-valued variables to optimize. Following convention, heuristic algorithms and a combination is used search to find efficient designs for the reduced groundwater model using various optimality criteria. The main goals are to introduce new design criteria and the concept of design efficiency for experimental design research in hydrology. The two criteria have good utility but interestingly, do not seem to have been implemented in hydrology. In addition, design efficiency is introduced. Design efficiency is a method to assess how robust a design is under a change of criteria. The latter is an important issue because the design criterion may be subjectively selected and it is well known that an optimal design can perform poorly under another criterion. It is thus desirable that the implemented design has relatively high efficiencies under a few criteria. As applications, two heuristic algorithms are used to find optimal designs for a small synthetic aquifer design problem and a design problem for a large-scale groundwater model and assess their robustness properties to other optimality criteria. The results show the proof of concept is workable for finding a more informed and efficient model-based design for a water resource study.

## 1. Introduction

With ever increasing demands being placed on groundwater aquifers, the needs to accurately model and understand behavioral properties of the aquifers are becoming more important. A common approach is to adopt a model-based approach and collect data to infer interesting features of the aquifers by estimating the model parameters, or some functions thereof. Unfortunately, accurate groundwater models are complex, high-dimensional and often have several physical and geographical constraints placed on the optimization problem so that obtaining the best possible estimates for the model parameters becomes challenging and computationally expensive. Many modern tools, such as Monte Carlo methods and global search algorithms used for optimization like genetic algorithms and particle swarm optimization, require hundreds, if not thousands of model calls. The full model sheds invaluable light on the behavior of groundwater but its complexity and the prohibitive computational expense required to analyze them frequently limit their usefulness in many applications. There is thus much research to find ways to solve the groundwater modeling and computation issues more efficiently. One approach is to use an efficient and robust design to collect data judiciously so that the estimated parameters are most accurate for a given cost.

As a concrete example of design issues that typically arise in such problems, consider the Owens Valley, north-east of Los Angeles, where the Los Angeles Department of Water and Power (LADWP) operates groundwater extraction wells to supply water for in-Valley use and for export to Los Angeles. Various appropriate methods are applied to avoid negatively impacting native plant species, meadow areas, and other habitats reliant on groundwater. In some areas, it is preferred that a minimum groundwater elevation be maintained; and in others, an optimal seepage flow is desired. To accomplish these goals, LADWP monitors the groundwater elevation at various locations in the Owens Valley through the use of groundwater monitoring wells. Deciding where to install these monitoring wells is carried out in a sequential manner and depends heavily on expert knowledge.

When a new groundwater extraction project is started and there is no good estimate of groundwater levels or an area of vegetation or habitat has been identified as being of concern, the general approach to choose a new observation well location is to rely on expert geologic knowledge to choose a good location between areas of concern and the nearest extraction wells. While effective, this sequential, local-optimization style approach does not guarantee an optimal distribution of observation wells. Artificial Intelligence coupled with a model-based approach to designing the study can offer multiple advantages. For example, given a set of observation wells, it is possible to obtain a bound on the uncertainty of the groundwater elevation. Another advantage is that multiple observation wells may be optimally located to maximize information and minimize redundant information at the same time. A third advantage is that the viability of alternate observation locations can be investigated.

However, a model-based approach requires specification of a plausible model, which in this case is a system of partial differential equations, along with adequately specified constraints and right initial conditions. Constraints can be geographical considerations like those above and they have to be properly formulated. The choice of an optimality criterion depends on the goal of the study and since different criterion leads to different designs, it is desirable to have a design robust to the design criterion. In addition, the design depends on the model and whether there are multiple goals in the study, and if so, are all the goals equally important. The upshot is that the implemented design should also be relatively robust to slight misspecifications in the model and be relatively efficient under the various competing goals. Thus there are multiple difficult design issues that need to be carefully considered upfront before the design is implemented. These design issues are certainly not unique in the design for monitoring wells but they have the distinguishing feature that the models are implicitly determined and the constrained optimization problem is much more complex and high-dimensional than those commonly encountered in a class on design of experiments. In particular, there are hundreds or thousands of integer-valued and continuous variables to be optimized.

This paper has a few aims: (a) provide a review of the statistical background in groundwater modeling problems and a brief literature review on groundwater modeling, (b) demonstrate the practical utility of a Galerkin method and a Proper Orthogonal Decomposition (POD) to simplify the design problems in groundwater modeling, (c) use metaheuristic algorithms to find various types of efficient designs for the reduced model, (d) propose two new design criteria, G- and I-optimality, for hydrology research, and (e) implement an efficient design robust to other optimality criteria. The latter two aims are the primary motivations as the two proposed criteria appear at least as relevant or more than currently used criteria in design research for water resources allocation problems and, the practically useful concept of design efficiency across criteria or models seem yet to be used in hydrology. Section 2 provides background on research in groundwater research with a focus on design issues for groundwater studies that includes current design methodologies, the use of POD, statistical terminology and optimality criteria. Section 3 discusses next steps after using POD to project the high-dimensional model space of the original groundwater model (referred to as the full model) into a lower-dimensional model space to create a reduced groundwater model. In particular, it has been shown that the reduced model can be solved several orders of magnitude faster than the full model and is able to accurately estimate the result from the full model [1]. Much research has been performed on applying POD model reduction in general, for example cavity flow [2], models of electromagnetism [3], and general linear systems [4] as well as specifically to applying POD to groundwater modeling. Vermeulen et al. [5] laid down the basic framework for applying POD to groundwater modeling on which Siade et al. [1] improved by developing a generalized reduction methodology and extension to nonlinear groundwater models [6]. Section 3 applies the methodology coupled with metaheuristic algorithms to two test cases and Section 4 concludes with a discussion.

## 2. Background

In general, the design of an observation network is formulated as an optimization problem whose optimality criterion is some measure of the useful information contained in a design’s information matrix. The formulation usually lends itself to a combinatorial optimization problem that is non-linear and non-convex. In the context of groundwater modeling, much research has attempted to overcome the challenges posed by these non-linear, non-convex, combinatorial optimization problems. Heuristic searches such as Genetic Algorithms (GAs) or Particle Swarm Optimization (PSO) searches have demonstrated their ability to search-out the solution to large-scale optimization problems that are difficult or impossible to solve with traditional mathematical programming techniques. GAs have been applied effectively to experimental design in groundwater modeling, for example in the areas of real-time optimization [7], groundwater management [8], groundwater contaminant monitoring [9]; however because these heuristic searches require computationally expensive model calls, many realistic experimental design applications are intractable.

### 2a. Reduced confined aquifer groundwater model

Recent research in the literature demonstrates methods to combat this computation complexity with model reduction techniques, such as a Galerkin method, along with a POD, which can be used to build a so-called reduced model which reduces the dimension of highly discretized groundwater models. These reduced models may be constructed to be independent of variations in forcing [10] or model parameter [11]. The reduced model is derived by expressing the governing equation for three-dimensional groundwater flow in a confined, anisotropic aquifer with the following partial differential equation (PDE) [12]:

$$\frac{\partial }{\partial x}\left(K_{x}\frac{\partial h}{\partial x}\right)+\frac{\partial }{\partial y}\left(K_{y}\frac{\partial h}{\partial y}\right)+\frac{\partial }{\partial z}\left(K_{z}\frac{\partial h}{\partial z}\right)-F=S_{s}\frac{\partial h}{\partial t}$$ (1)

with initial and boundary conditions

$$\begin{array}{c} h(x,y,z)=f_{1}(x,y,z)\\ h(x,y,z,t)=f_{2}(x,y,z,t),\;(x,y,z,t)\in (\Gamma_{1})\\ q_{n}(x,y,z,t)=f_{3}(x,y,z,t),\;(x,y,z,t)\in (\Gamma_{2}) \end{array}$$

where *h* is the hydraulic head [L] (the height of the sub-surface water table above a references elevation); *K*_*x*_, *K*_*y*_, and *K*_*z*_ are the hydraulic conductivities in the *x*, *y*, and *z* directions [L/T] (the speed with which water moves through the medium); *S*_*s*_ is the specific storage [L^-1^] (the volume of water that will be released due to a decrease in *h*); *F* is the specific volumetric pumping rate [T^-1^] (the volume of water being extracted); *q*_*n*_ is the specific discharge normal to the flux boundary (Γ_2_) [T^-1^] (the volume of water flowing out of the model space); Γ_1_ is the fixed head boundary (a Dirichlet boundary such as a lake which maintains *h* along it at a fixed level); *f*_1_,*f*_2_ and *f*_3_ are known functions; L denotes the length unit (meters, feet, etc.) and T denotes the time unit (days, hours, etc.). The governing PDE in Eq (1) is then discretized through finite difference or finite element approximations in space and finite difference in time to produce a set of linear equations for each time step:

A(k)h=b

where $A(k)\in R^{N_{n}\;x\;N_{n}}$ contains the model parameters and the initial boundary conditions, $b\in R^{N_{n}}$contains the forcing (groundwater pumping, recharge, etc.), *N*_*n*_ is the total number of nodes used to discretize the aquifer, and ***k*** is a vector of hydraulic conductivities. In order to accurately model most real-world groundwater aquifers which may cover hundreds of square kilometers, many nodes are needed, thus *N*_*n*_ will be very large. This is important because *N*_*n*_ defines the dimension of the groundwater model and when *N*_*n*_ is large, solving (1) is computationally expensive. POD may be used to reduce this model by constructing some matrix $\text{P}\in R^{N_{n}xn_{p}}$such that the projection

$$P^{T}A(k)Pr=P^{T}b$$

produces a reduced model with dimension *n*_*p*_ and state vector $\text{r}\in R^{n_{p}}$ where ***h*** ≈ ***Pr*** and is much less computationally expensive than the full model to solve because *n*_*p*_ << *N*_*n*_. The reduced model may then be coupled with a heuristic search to perform a combinatorial search in a reduced space. This enables us to investigate how to best apply experimental design techniques to groundwater problems that were previously intractable.

### 2b. Experimental design and model

An experimental design is a sampling scheme that dictates how observations are collected for the study. Designs may be model based or not and the study objective may be single or multiple objectives. Typically objectives for a study are parameter estimation and prediction or more specialized objective, such as extrapolation at low doses in toxicology studies. An optimal design is a plan to attain the objective most efficiently. For example, if the objective is to estimate model parameters, an optimal design provides the most accurate estimates at minimal cost given a fixed amount of resources. Objectives are sometimes called design criterion, goal or goodness of measure and they can vary considerably. For example, it can include goals like minimizing some measure of error in the specification of the proposed model or minimizing the maximal predictive variance across a pre-specified region of the design space.

Selecting an appropriate design criterion can be subjective but is an important decision. Each optimal design has its pros and cons and some can suffer a noticeably drop in efficiency under another criterion or are more robust to model misspecifications. In the context of groundwater models, the choice of the sampling scheme is frequently in the choice of an optimal observation network. This is tantamount to making best choices for the spatial and temporal distribution of measurements of groundwater head. It is possible to employ multi-objective optimization, e.g. coupling a classic optimality criterion with a metric of the distance between observations [13] and employ statistical methods such as those in Cook and Wong [14] and, Clyde and Chaloner [15]. The former considered dual objective optimal design problems for linear models and the latter extended the work to finding multiple-objective optimal designs for nonlinear models. However, many times a single objective optimization is used, with A- or D- optimality being the most common criterion with uses including parameter estimation for groundwater modeling [16], column outflow [17], dispersion equations [18], and transport equations [19]. It has also been used for model discrimination [20] and designing pump tests [21] and tracer tests [22]. Both of these two criteria offer different benefits and drawbacks. This reality highlights the fact that the “best” answer might be a compromise among the optimality criteria rather than a single result notwithstanding that this would result in prohibitively expensive to computation fora multi-objective design. The challenge, then, is to find the best-feasible solution, i.e. one that is not prohibitively expensive to find. This suggests that it is helpful to have more design options. In the next section, common design criteria are reviewed and two new and useful design criteria are proposed for research work in hydrology. The experimental designs are model-based and a general linear model is used to approximate the solution found by solving Eq (1). The simplified model is given by

$$s=J_{d}\theta ,$$ (2)

where ***s*** ∈ *R*^*m*^ is the state vector, ***θ*** ∈ *R*^*n*^ is the vector of model parameters and ***J***_***d***_ ∈ *R*^*m x n*^ is the sensitivity matrix (or the Jacobian matrix) defined by

$$J_{d}=\left(\begin{array}{ccc} \frac{\partial s_{1}}{\partial \theta_{1}} & \ldots & \frac{\partial s_{1}}{\partial \theta_{n}}\\ \vdots & \ddots & \vdots \\ \frac{\partial s_{m}}{\partial \theta_{1}} & \cdots & \frac{\partial s_{m}}{\partial \theta_{n}} \end{array}\right).$$ (3)

Here *s*_*i*_ is the *i*^*th*^ element in ***s*** and *θ*_*j*_ is the *j*^*th*^ element in ***θ***, and $\frac{\partial s_{i}}{\partial \theta_{j}}$ is the sensitivity of *s*_*i*_ with respect to changes in *θ*_*j*_. Eq (2) is a surrogate model for some system model–in this case, a groundwater model. To set-up the experimental design problem the elements of ***J***_***d***_ are formed from the groundwater model of which information is sought. Three different methods can be used to calculate the sensitivities in ***J***_***d***_: (1) the parameter perturbation method, (2) the sensitivity equation method, or (3) the adjoint state method [23]. The parameter perturbation method has the following general form:

$$\frac{\partial s_{i}}{\partial \theta_{j}}\approx \frac{\Delta s_{i}}{\Delta \theta_{j}}=\frac{s_{i}(\theta +{\Delta \theta }_{j})-s_{i}(\theta )}{\Delta \theta_{j}}$$ (4)

where Δ*θ*_*j*_ is a small change of the *j*^*th*^ parameter (called the perturbation of *θ*_*j*_) [24]. There are two general requirements for using Eq (4) to make accurate estimates of $\frac{\partial s_{i}}{\partial \theta_{j}}$. First, ***θ*** must be close to the true parameter values, and second, Δ*θ*_*j*_ << *θ*_*j*_.

### 2c. Optimality criteria

When the objective or objectives of a study are clearly elicited, data should be judiciously collected to attain the objective maximally. For example, if the interest is to estimate parameters in a given model, data should be collected so that the parameters are estimated with maximum precision at minimal cost. To this end, a given model is assumed and a stated objective is expressed as a mathematical function of the design variables with the goal being to optimize the objective function by choice of the settings of the design variables. In the context of the given examples, the design variables are the locations of the wells to be constructed in the region of interest. The objective function is mathematically formulated and is usually called the design criterion. There are two general classes of information being sought: (1) Information about the model parameters (***θ***) and (2) Information about the model’s predictions of the state vector (***s***).

From classical experimental designs, there are several design criteria for estimating model parameters or the response surface, wholly or partially. Table 1 lists 5 common optimality criteria in the statistical literature [25].

**Table 1:** Optimality criteria.

Criteria	Information Class	Objective Function ɸ(*ω*)	Description
A	***θ***	$\begin{array}{c} \max\\ \omega \end{array}\left(trace\left(F\right)\right)$	Maximizes total information
E	***θ***	$\begin{array}{c} \max\\ \omega \end{array}\left(\begin{array}{c} \min\\ \lambda_{i} \end{array}\left(F\right)\right)$	Maximizes unique information
D	***θ***	$\begin{array}{c} \max\\ \omega \end{array}\left(\det\left(F\right)\right)$	Maximizes uncorrelated information
G	***s***	$\begin{array}{c} \min\\ \omega \end{array}\left(\begin{array}{c} \max\\ i \end{array}\left(j_{d,i}\left(F^{-1}\right){\left(j_{d,i}\right)}^{T}\right)\right)$	Minimizes the maximum prediction error
I	***s***	$\begin{array}{c} \min\\ \omega \end{array}\left(\begin{array}{c} \text{mean}\\ \forall i \end{array}\left(j_{d,i}\left(F^{-1}\right){\left(j_{d,i}\right)}^{T}\right)\right)$	Minimizes the average prediction error

Given a sampling strategy, or equivalently, a design *ω*, defined on a user-specified experimental region, the matrix F in the table is given by

$$F=J_{d}{}^{T}J_{d}$$ (5)

where ***j***_*d*,*i*_ is the *i*^*th*^ row in ***J***_***d***_, *λ*_*i*_ is the *i*^*th*^ eigenvalue of ***F***, *ω* is some sampling strategy, and ***J***_***d***_ is design matrix constructed from the design *ω* in (3). It is assumed that 1) a least-squares error criterion is used for parameter estimation and 2) the observation errors are uncorrelated and have equal variance. The matrix ***F*** is the inverse of the covariance matrix of the estimated parameters [26]. Among the listed design criteria, the third design criterion is most frequently used in hydrology and in other disciplines as well. The resulting optimal design is called D-optimal and it minimizes the volume of the confidence ellipsoid for the unknown model parameters. A-optimality has a similar goal of estimating the model parameters as precisely as possible; however in hydrology it is defined differently as the A-optimality used in the statistical literature. The latter finds design points that minimize the volume of the confidence ellipsoid by minimizing the sum of the lengths of its principal axes. Both criteria may be viewed as an approximation to one another.

The above criteria can be formulated in terms of the eigenvalues of the matrix ***F***. For example, it can be shown that D and A-optimality can be expressed as the product and average of all the eigenvalues of ***F***, respectively. Likewise, E- optimality finds a design that maximizes the minimum eigenvalue of the information matrix. An E-optimal design may be used for estimating model parameters or for guaranteeing the highest power for conducting an omnibus test whether explanatory variables are helpful in a linear regression model. Among all criteria in Table 1, Pukelsheim [27] noted that the statistical justification for E-optimality seems the weakest.

Unlike D-, A- and E-optimality, G- and I-optimality have not been applied widely to groundwater modeling even though they have sound statistical justifications. G-optimal designs estimate the entire response surface by minimizing the largest variance of the fitted responses across the design space. Like E-optimality, the G-optimality is not a differentiable function and so properties of these two optimal designs are more difficult to study mathematically. There seems to be no known algorithms that are guaranteed to converge to the G- and E-optimal designs and generally specialized algorithms are used to find them even when the model is linear, see for example Rodriguez, el at. [28] and Hernandez and Nachtsheim [29], respectively. I-optimality is an averaging criterion and allows the fitted responses to be estimated over different parts of the experimental region with user-selected emphasis. As an example, suppose a nonlinear model has a mean response modeled by ***f***(*x*,*θ*), the G-optimality criterion is to find a design to minimize the maximum value of approximate value of the asymptotic variance of fitted response at *x*, i.e. ***v***(*x*,*θ*) = ***g***^T^(*x*,*θ*)***F***^-1^***g***(*x*,*θ*), where ***g***(*x*,*θ*) is the derivative of ***f***(*x*,*θ*) with respect to *x* and *x* ranges over the entire the experimental region. The I-optimality criterion is formulated as ∫***v***(*x*, *θ*)*μ*(*dx*), where *μ*(*dx*) is the weighting measure across the experimental region. It is thus a weighted average of the variance of the fitted response across the experiment region with greater weights for the more interesting parts. If *μ*(*dx*) is degenerate and puts all its mass at a single point, then the design problem is an interpolation or extrapolation, depending whether the point is inside or outside the experimental region. Further mathematical properties of these criteria and their interpretation can be found in design monographs in statistics, such as, Berger and Wong [30], Pukelsheim [27] and Silvey [31]. The first monograph covers introductory material and aimed at the applied researchers, the second has a high level of mathematical contents and the third is at a somewhat intermediate level between the first two. Table 1 displays the discrete versions of G- and I-optimality, where the range of *i* may be limited, i.e. the minimization can be carried-out over some subset of ***s*** that is of particular interest or over all the elements in ***s***. In Table 1, λ_i_ is the i^th^ eigenvalue of the matrix ***F*** and the outer optimization of the design ω is among all designs in the experimental region.

Once a model is postulated and an appropriate optimality criterion is selected, the experimental design problem may generally be formulated as a constrained optimization problem, i.e. find a design to optimize ɸ(***ω***) among all designs ***ω*** in the user-selected set ***Ω*** contains all possible sampling locations that satisfy the imposed constraints. The constraints typically include limitations on the size (number of observation wells), location, or distribution of ***ω***, along with possibly non-linear constraints that arise from financial or geographic considerations mentioned above.

### 2d. Optimal efficiency

In the seminal work on experimental design, Kiefer [32] argued that a design that is optimal in one measure can be close to optimal with respect to other measures even the two criteria can differently motivated. For example, he showed that D and G-optimal designs are the same when the model is homoscedastic even though the two criteria have very different and useful goals. However, there are numerous examples in the statistical literature that show an optimal design constructed under one criterion can perform poorly under another criterion. The practical implication is that one should implement a robust design that performs well under two or more design criteria. There is virtually no design research in the field of groundwater research that uses this concept of design efficiency in evaluating the worth of a design. This paper uses two experimental design problems and shows that this proof of concept is also workable in hydrology research.

More generally, the performance of a design is compared with another design under the same or a different criterion. Let ɸ(*ω*) be the design criterion and suppose a design ω* that minimizes its value among all designs. The ϕ-efficiency of a design *ω* is defined by the ratio

$${\text{E}}_{\phi }(\omega )=\phi (\omega^{*})/\phi (\omega )$$

Clearly, the efficiency of any design and any criterion, E_ɸ_, has a value between 0 and 1. If the criterion is G-optimality and the ratio is 0.5, the practical implementation is that the design *ω* has to be replicated twice to do as well as the optimum ω* in terms of the G-optimality criterion. The ratio can also be used to compare the competitiveness of two optimality criteria. For instance, to assess how the G-optimal design performs under the I-optimality criterion and vice versa, (5) measures their relative efficiencies. This ratio can convey, for example, how much the G-information is contained in an I-optimal design. Wong [33] provides further examples on how efficiencies of a design change across models and optimality criteria, and details how the above ratio needs to be amended for some criterion, like D-optimality, to maintain the appeal of the practical interprtation.

### 2e. Evaluation challenges

In general, for small-scale problems, A-optimal designs may be found through linear programming, and D-, E-, and I-optimal designs may be found using convex optimization tools. G-optimal designs, however, are not linear or convex and are generally difficult to find even for small-scale problems. In the context of experimental design problems for developing an optimal observation well network for a groundwater model, solving such optimization problems is even harder. Often these problems are non-convex, non-differentiable, and require a combinatorial search to solve. For a realistically-scaled groundwater model, the combinatorial search would be infeasible to solve with traditional mathematical programming techniques because of the hundreds or thousands of nodes in the model.

Nature-inspired metaheuristic algorithms are essentially assumptions free and are increasingly used to overcome all types of challenging optimization problems, including for the problems described herein [34]. They are fast and easy to implement. Genetic Algorithm (GA) and Particle Swarm Optimization (PSO) are examples of metaheuristic algorithms. GA utilizes breading and mutation to search a solution space [35] and PSO searches in the solution space by deploying sets of particles that utilize position and velocities to update the best candidate solution [36]. The algorithms come with different motivations and so, as expected, they have their own unique advantages and disadvantages. Utilizing velocities allows PSOs to converge quickly but at the cost of an increased likelihood of becoming trapped in a local optimum. On the other hand, a GA sacrifices speed to take advantage of the randomness of its mutations to attempt to avoid being trapped in local optima [37]. All metaheuristic algorithms have tuning parameters and a common challenge working with these algorithms is to find appropriate values for them so that the algorithms perform well.

Metaheuristic algorithms generally do not have proofs of convergence and so they do not guarantee convergence to the global optimum. They are stochastic components in these algorithms and consequently, they will not necessarily produce the same design with repeated runs. Nevertheless, it is widely reported that they frequently produce the optimum or solutions close to the optimum, which explains their popularity [38, 39]. The researchers experience is similar; GA, PSO and modern nature-inspired metaheuristic algorithms, such as swarm-based techniques [40, 41] Imperialist Competitive Algorithm [42], Differential Evolutionary [43, 44] and Competitive Swarm Optimizer [45] can also produce highly efficient designs for complicated nonlinear models with or without random effects. They include finding high-dimensional optimal supersaturated designs, more flexible adaptive two-stage Phase II designs [41] and Bayesian optimal designs [42] using adaptive cubature for models with possibly multiple interacting factors and some factors are discrete [43] or continuous [46] or random [45]. The searches in the current research have the additional challenge that an inordinately large number of model calls are required, possibly rendering the search ineffective. However, past research has demonstrated that this can be overcome through methods such as model reduction [10, 11].

From a computational standpoint, finding an A- or E-optimal design ***ω*** is relatively easier than searching for D-, G-, or I-optimal designs. Computing the determinant (det(•)) of a general matrix will be unstable for a nearly singular matrix (i.e. det(•) ≈ 0). For this reason utilizing the determinant in an algorithm is discouraged [47]. A method that can be used to stabilize the calculation of the determinant takes advantage of the fact that the determinant of a matrix is the product of all the eigenvalues of that matrix. One could include a heuristic to declare the determinant equal to zero if a small enough eigenvalue is observed.

An additional challenge that optimal observation network heuristic searches face when utilizing D-optimality is that the objective function surface tends to be very flat, making it very difficult to find a good search direction. A way to overcome this challenge is to artificially exaggerate the objective function surface features to assist the algorithm to find a good search direction. Since det(*c****A***) = *c*^*n*^ det(***A***) for any square matrix (***A***) and constant *c*, *c* is chosen such that O(det(*c****F***)) ≈ 1, where O(•) is the order of magnitude. This simple procedure ensures that small fluctuations in the objective function surface are magnified so that they are not missed by a heuristic search due to round-off error. Once the heuristic algorithm has searched-out the optimal solution, *c*^*n*^ may then be divided-out to obtain the true value of det(***F***).

To evaluate G- or I-optimality, there are potential numerical stability issues because ***F*** has to be inverted. To this end, singular value decomposition is applied to Eq (5) obtaining

$$F^{-1}=J_{d}^{-1}{\left(J_{d}^{T}\right)}^{-1}=J_{d}^{-1}{\left({\text{J}}_{d}^{-1}\right)}^{T},$$

where ***J***_***d***_ = ***UΣV***^***T***^, ***U*** is a matrix containing the left singular vectors, ***Σ*** is a diagonal matrix containing the singular values (*σ*_*i*_), and ***V*** is a matrix containing the right singular vectors. Consequently, $J_{d}^{-1}=V\Sigma^{-1}U^{T}$,

$$\begin{array}{c} j_{d,i}\left(F^{-1}\right){\left(j_{d,i}\right)}^{T}=j_{d,i}\left(V\Sigma^{-1}U^{T}U\Sigma^{-1}V^{T}\right){\left(j_{d,i}\right)}^{T}\\ =\left(j_{d,i}V\right)\Sigma^{-2}{\left(j_{d,i}V\right)}^{T} \end{array}$$

and the values of G- and I-optimality of a design ***ω*** can be evaluated stably.

## 3. Optimal efficiency study

**Fig 1:**
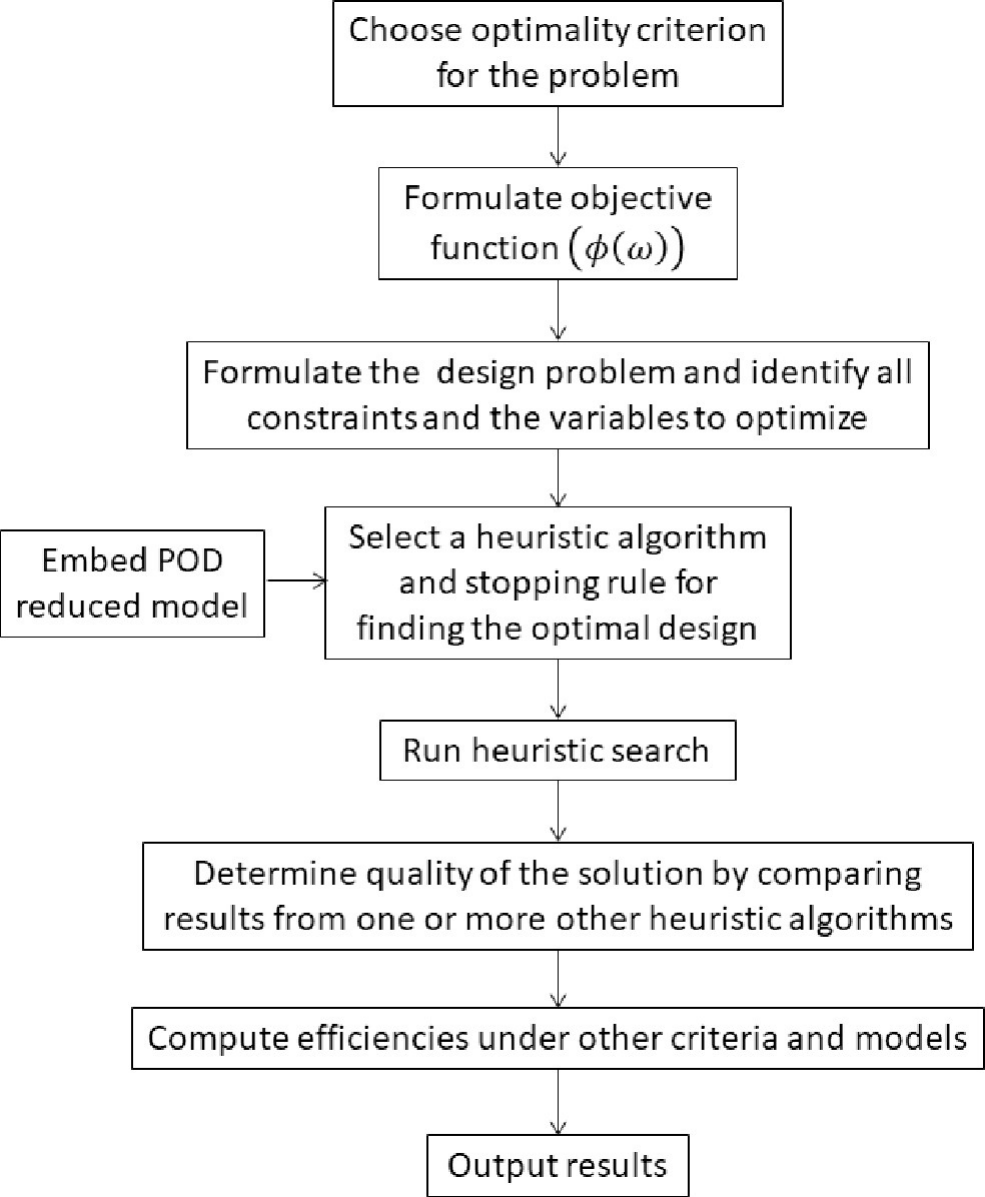
Steps to carry out an optimal efficiency study.

With each of these elements in place, a course of action is constructed to evaluate the variation in optimal efficiencies within a groundwater model. The flowchart (Fig 1) describes the steps to carry out an optimal efficiency study. The optimality criterion should be chosen with the scientific question in mind and the mathematical optimization problem is then carefully formulated. Even though it may be time consuming, it is helpful to run one or more appropriately selected metaheuristic algorithms to confirm optimality of the design before its sought robustness properties are evaluated.

## 4. Test cases and results

To demonstrate the method study two synthetic, two-dimensional test cases are developed. Though synthetic, these test cases fit two categories of model testing. The first test case is a small-scale test case that is easily reproducible and carried out without the need for specialized software or hardware. A model of this category allows for easy, independent analysis of the methodology proposed. The second test case is a large-scale test case, representative of a realistic model that would is encountered in groundwater modeling. This test case demonstrates the real-world applicability of the methods being proposed.

### 4a. Small-scale two-dimensional test case

**Fig 2:**
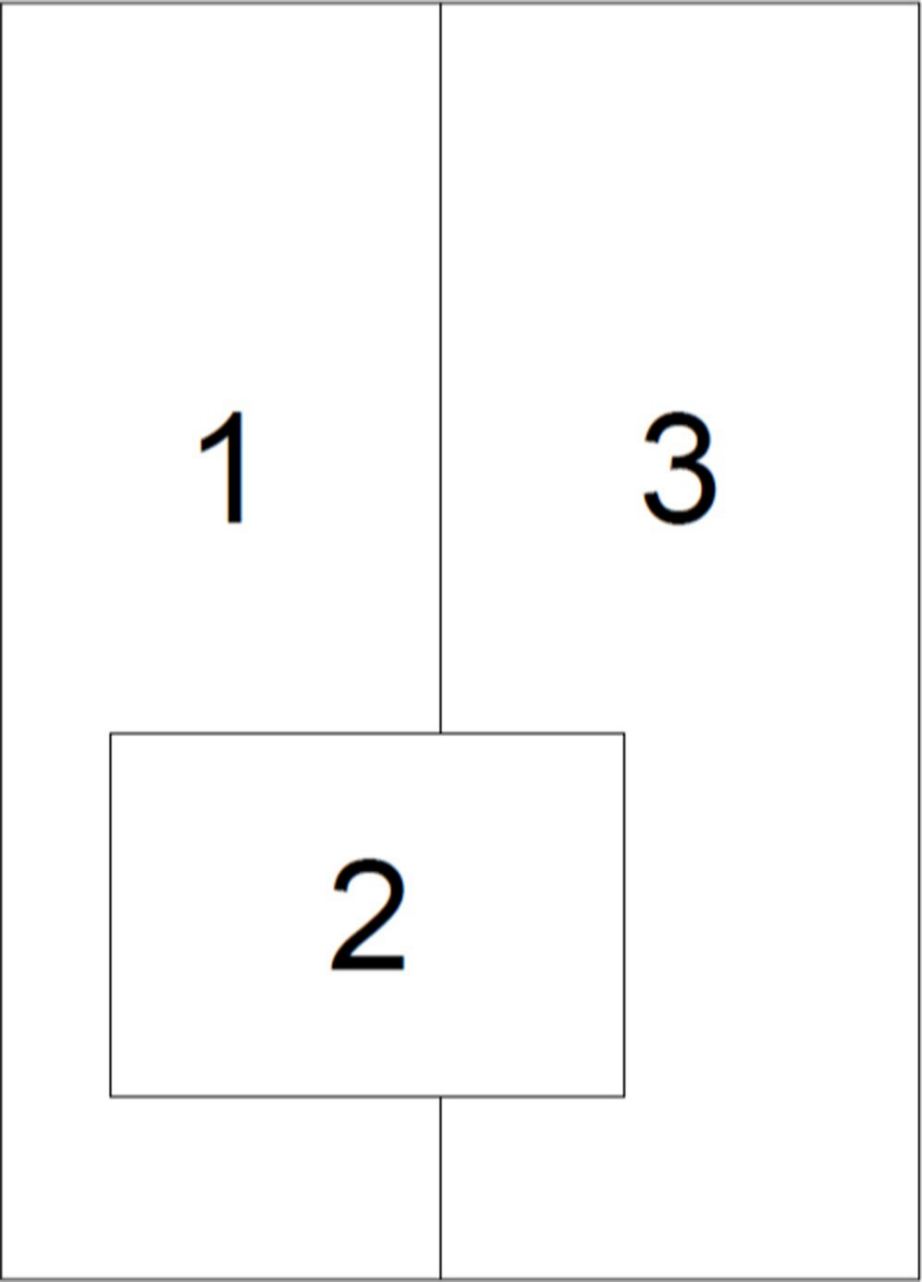
Small-scale test case hydrologic zones.

The first test case is synthetic, confined aquifer with dimensions of 25*m x* 35*m* and is divided into three hydrologic zones (see Fig 2) with varying hydrologic properties. Table 1 (Table 2, right?) shows the hydrologic properties of the aquifer. Note that an unusually large value of specific storage is assumed to speed-up the simulation (i.e. decrease the time required to reach steady state). Constant-head conditions are imposed on the north and south boundaries and no-flow conditions apply to the east and west boundaries.

**Table 2:** Small-scale test case hydrologic properties.

Zone	Specific Storage (*m*^-1^)	Depth (*m*)
1	1	20
2	1	15
3	1	22

**Fig 3:**
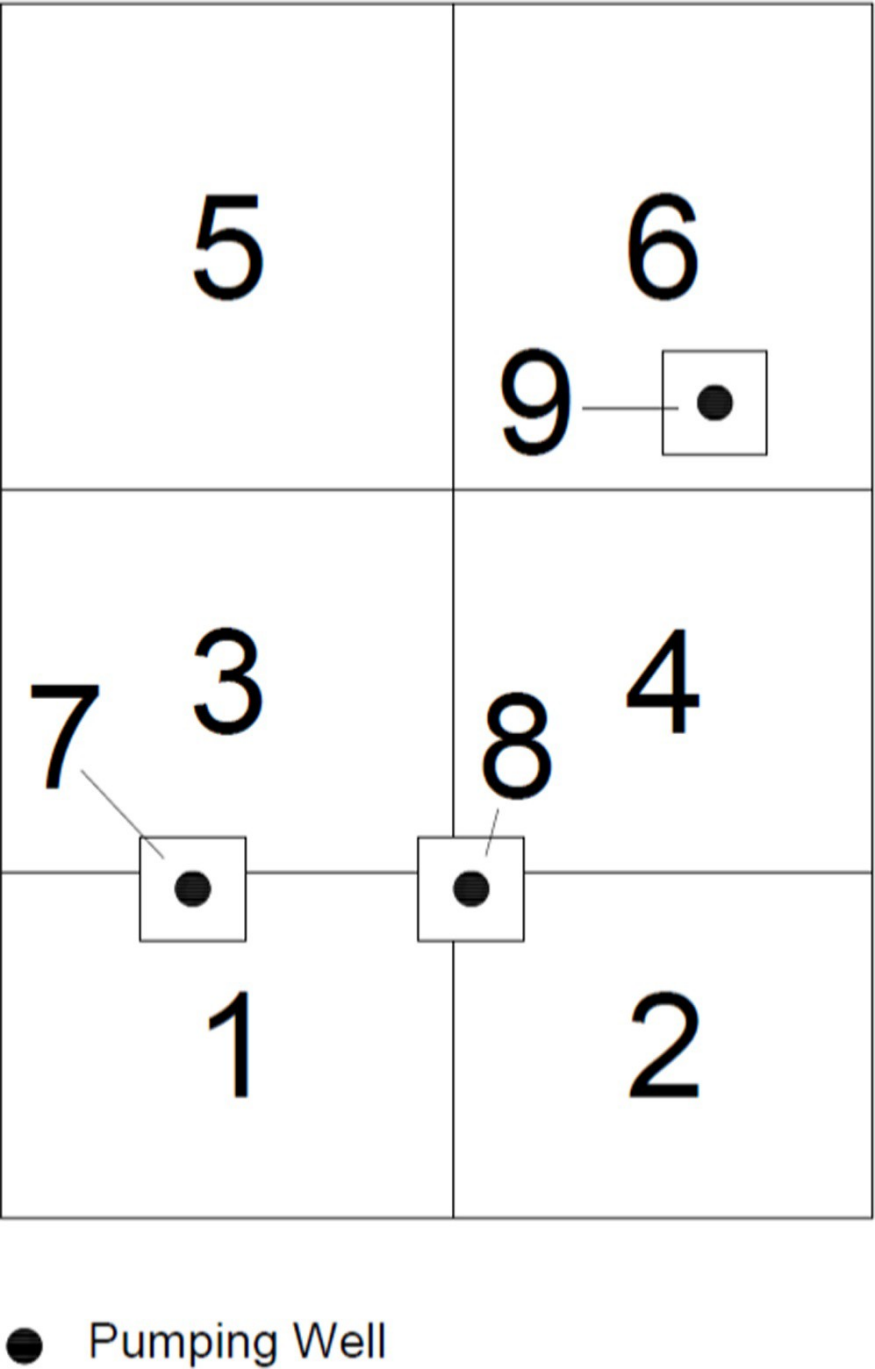
Small-scale test case experimental design setup.

The hydraulic conductivities in this aquifer are assumed to be unknown, so the experimental design seeks to gain information on the hydraulic conductivities in each of the zones. The aquifer is divided into nine observation/pumping zones (Fig 3). Zones 1–6 are set as observation zones and zones 7–9 are set as pumping zones. One set of constraints that is placed on the experimental design is that at most one observation well may be placed in an observation zone and no observation wells may be placed in a pumping zone.

Since ***J***_***d***_ depends on hydraulic conductivity, a robust experimental design is performed for the worst case scenario [11] assuming the hydraulic conductivity in each of the three hydrologic zones is in the range [0.1*m*/*day*, 20.0*m*/*day*]. The technique outlined by Ushijima and Yeh [11] is followed and the experimental design is solved over the set formed by discretizing the range of each zone into three levels, then forming all combinations of levels of zonal hydraulic conductivity. In addition to the constraints on number and location of observation wells with respect to the observation/pumping zones, the way the results vary with respect to changes in the total number of observation wells is explored by constraining the total number of observation wells, then varying that constraint over the range interval, [1,6]. In order to observe how differences in heuristic searches may affect the results, for each of the optimality criteria list in Table 1 both a GA [35] and a PSO [36] is used to find the robust one-observation-well network (one-well design) up through the six-observation-well network (six-well design). Table 3 shows the tuning parameters used for the heuristic algorithms. The default values provided by Wall and Pedersen were used for the two algorithms since our focus is not on the performance of the algorithms themselves. The developers of the particular algorithms note that although the algorithms are tuned for some circumstances, they are of course not generally tuned and the algorithms performances could be improved by applying appropriate parameter tuning techniques.

**Table 3:** Heuristic algorithm parameters.

GA Parameter	PSO Parameters
Number of generations: 250	Swarm size: 148
Crossover probability: 0.9	Inertia weight: -0.046644
Mutation probability: 0.01	Agent’s weight: 2.882152
Population size: 30	Swarm weight = 1.857463
Number of populations: 10	Termination: convergence for not less than 2000 generations
Replacement percentage: 0.25	
Elitism: TRUE	
Number of offspring: 2	
Migration percentage: 0.1	
Migration number: 5	
Termination: convergence for not less than 2000 generations	

The results found by the algorithms are tied closely to the specific experimental design setup, thus precluding absolute general statements, but some interesting observations may be made.

**Fig 4:**
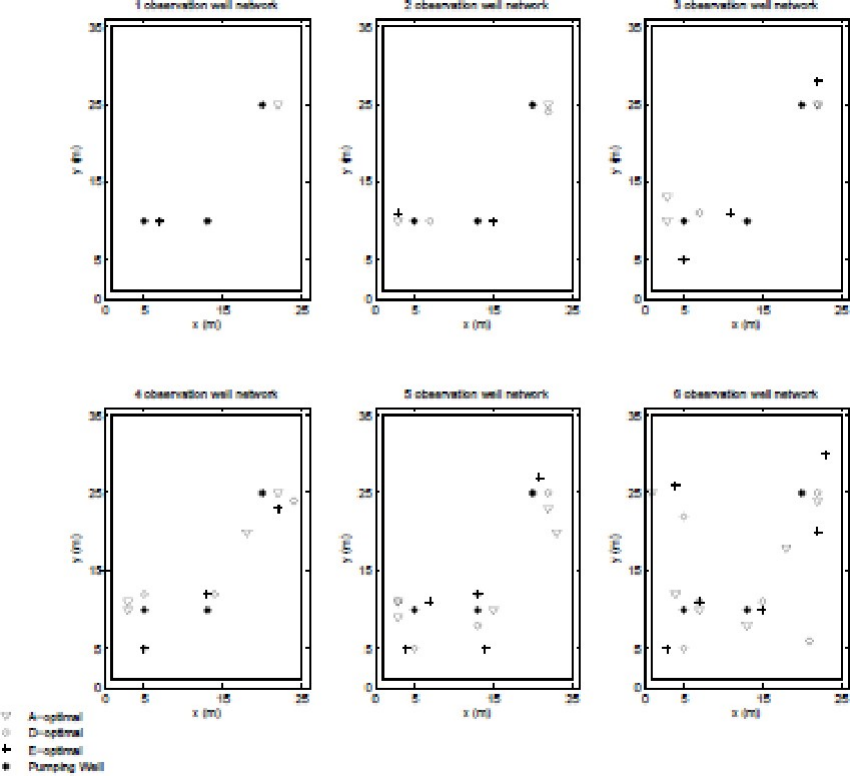
A-, D-, and E-optimal designs.

**Fig 5:**
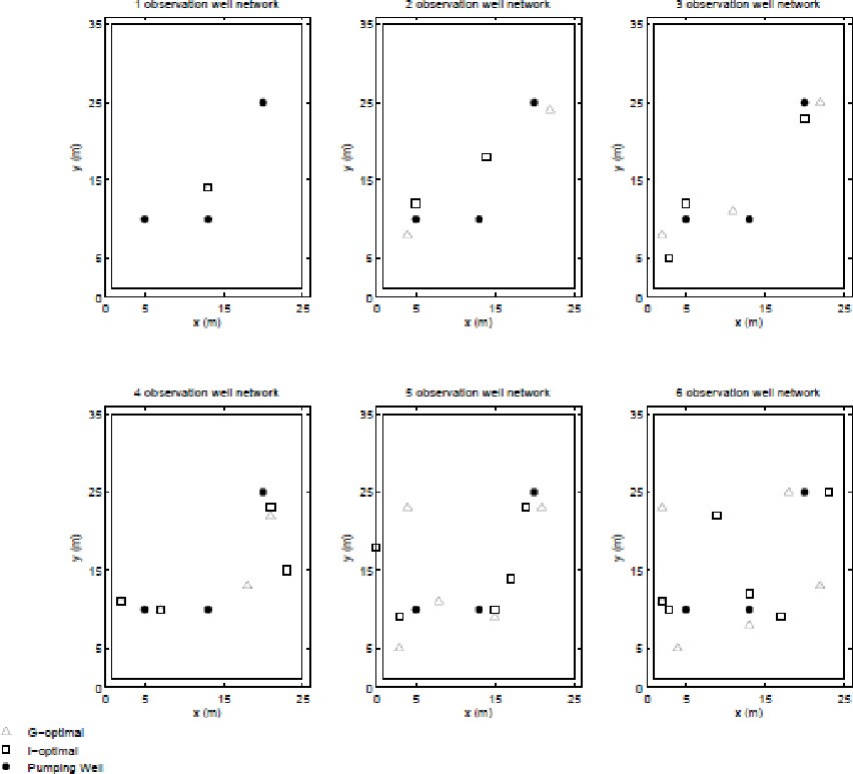
G- and I-optimal designs.

First, as would be expected, the GA and PSO converged to similar but different results in terms of both spatial distributions of the observation wells and objective function score. In general the scores were within O(1%) of each other but for this test case, the PSO outperformed the GA (i.e. produced more optimal results) for smaller designs (i.e. those with smaller observation well networks) but the GA outperformed the PSO for larger designs. From these two sets of results, the best (based on objective function score) designs were compiled into one set and are shown in Figs 4 and 5.

In the rest of the paper, the designs found by the algorithm are referred to as a ɸ –design. For example, an A-design is one found by the algorithm under the A-optimality criterion. The rationale is that metaheuristic algorithms do not guarantee that they will find the optimal design. However, repeated runs of the algorithm will frequently do so or find one with very high efficiency, which likely will suffice in practice. For this reason, the terms ɸ-design and ɸ-optimal design are sometimes used interchangeably.

Figs 4 and 5 show that the spatial distribution of observation well for the various designs are consistent with what would be expected from each of the criteria. The A-designs (i.e. the designs to which the GA and PSO converged over the A-solution space) grouped their observation wells close to pumping wells regardless of whether or not the information gained would be redundant; the D-designs tended to spread-out the observation wells the most, consistent with minimizing covariance; and the E- designs spread-out the observation wells evenly, consistent with maximizing unique information. Though it is harder to predict how I- and G-designs will behave individually but it may be guessed that both designs would have similar spatial distribution as both designs seek to gain the maximal of the same type of information. As may be seen in Figs 4 and 5, this assumption holds for this test case.

**Fig 6:**
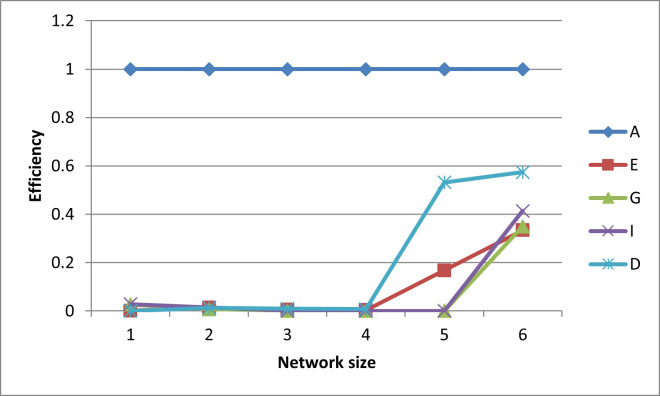
A- efficiencies of various optimal designs for the first case study.

**Fig 7:**
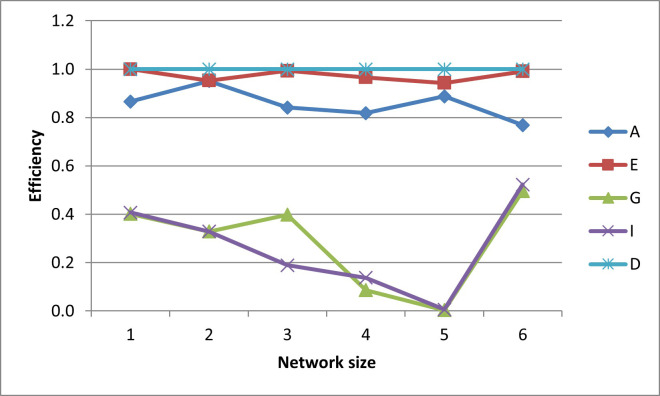
D-efficiencies of various optimal designs for the first case study.

**Fig 8:**
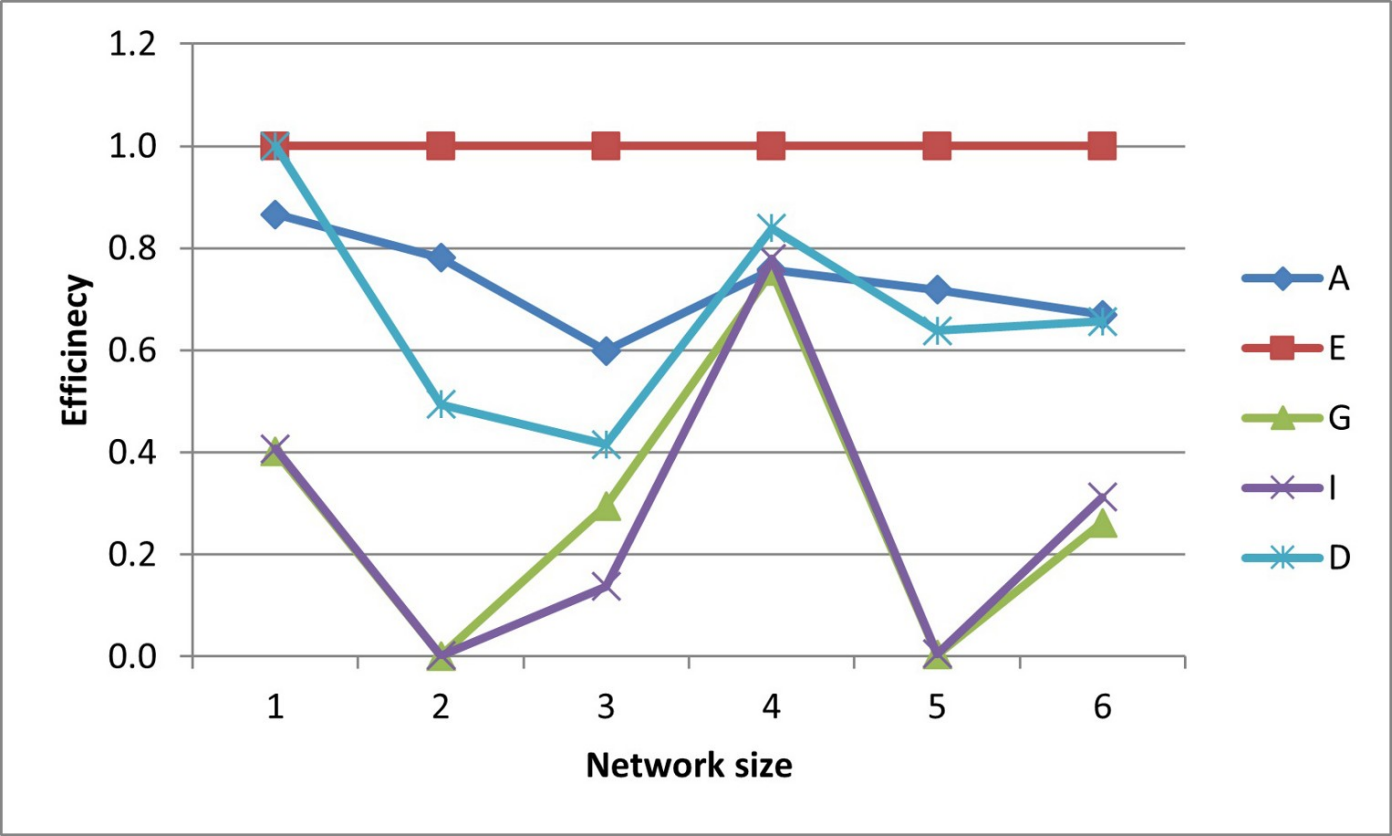
E-efficiencies of various optimal designs for the first case study.

**Fig 9:**
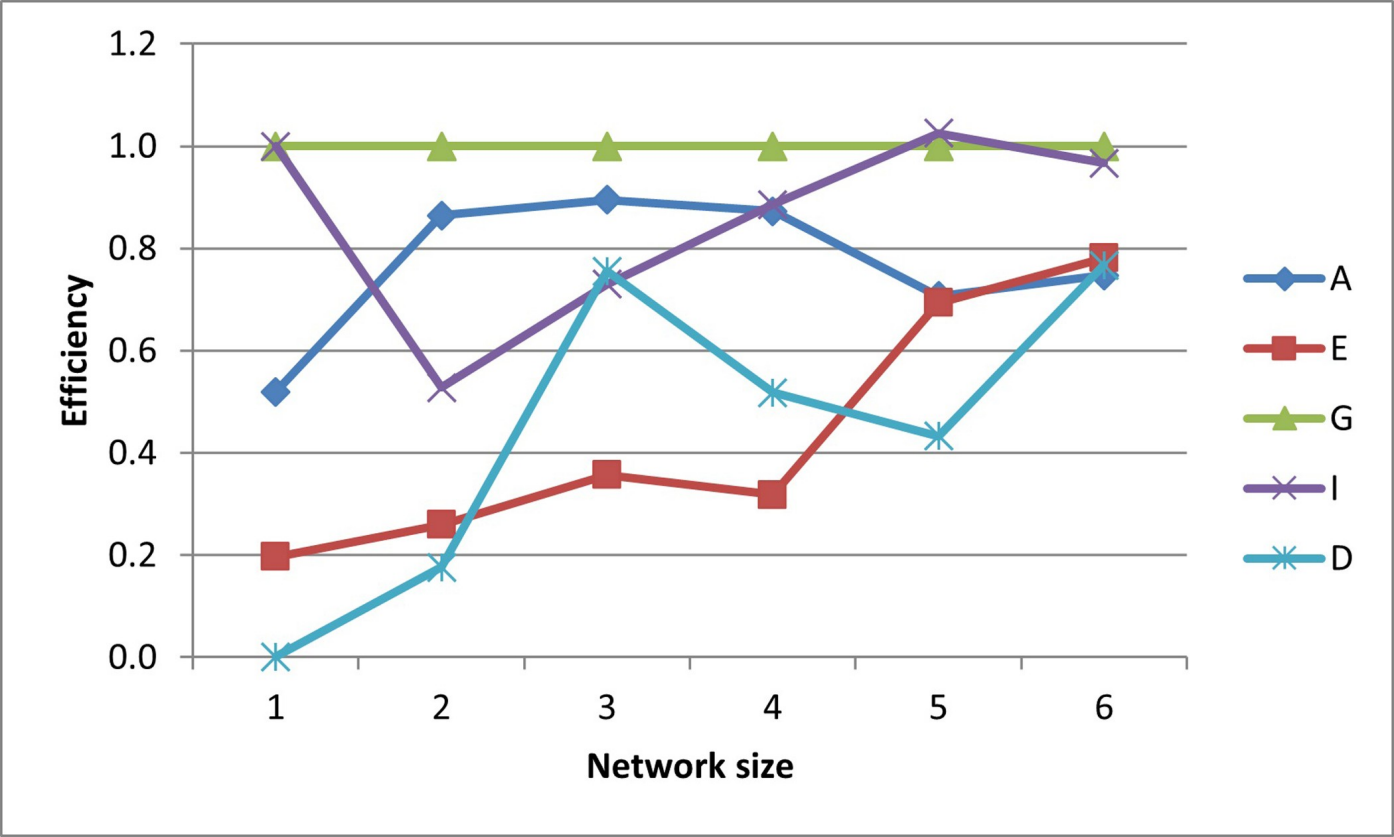
G-efficiencies of various optimal designs for the first case study.

**Fig 10:**
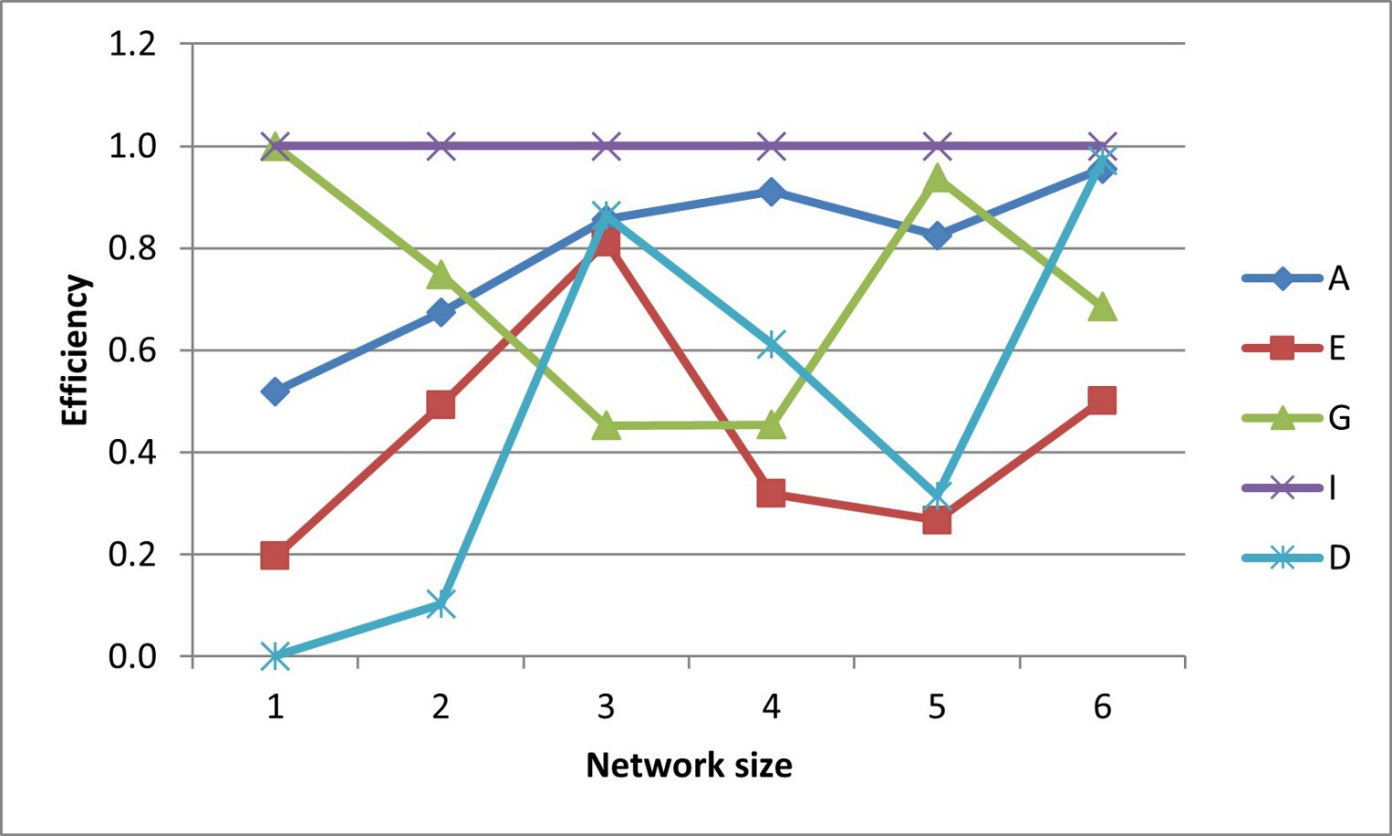
I-efficiencies of various optimal designs for the first case study.

After plotting-out the observation well locations, the efficiencies for these designs are graphed in Figs 6–10.

In this test case, note that all the designs are fairly A-optimal (i.e. have high A-efficiencies) but for the most part, the A-designs did not have high non-criterion efficiencies. As might be expected, the E- and D-designs are fairly optimal with respect to each other, as are the G- and I-designs. Taking a higher-level view, the prediction-designs (G- and I-designs) are more optimal with respect to the other prediction-design than they are to parameter-designs (A-, E-, and D-optimality). The same is observed for the parameter-designs, as they are more optimal with respect to the other parameter-designs than they are with respect to the prediction-designs. While analyzing the results, the general weakness of heuristic searches becomes apparent, as both the GA and PSO converge to sub-optimal solutions. This becomes obvious when the non-criterion efficiencies are calculated and it is clear that the GA-one-, three-, and four-well D-designs and the four-well G-design had E-efficiencies greater than 100%, i.e. these designs are more E-optimal than the E-designs of the same size. In addition, the five- and six-well G-designs have I-efficiencies greater than 100%. Thus it is concluded that the GA became trapped in sub-optimal solutions while searching the E- and I-solution spaces. Based on efficiency, it is also concluded that the PSO was trapped in a sub-optimal solution while searching for the five-well I-design. Although neither the GA nor the PSO are able to converge to the best observed location, both five-well designs were close approximations of the best observed location such that the I-efficiencies of the five-well G-design were 103% and 107%, respectively. Based on the “best” criterion, the GA-five-well I-design was included in the best set (Fig 9).

### 4b. Large-scale test case

**Fig 11:**
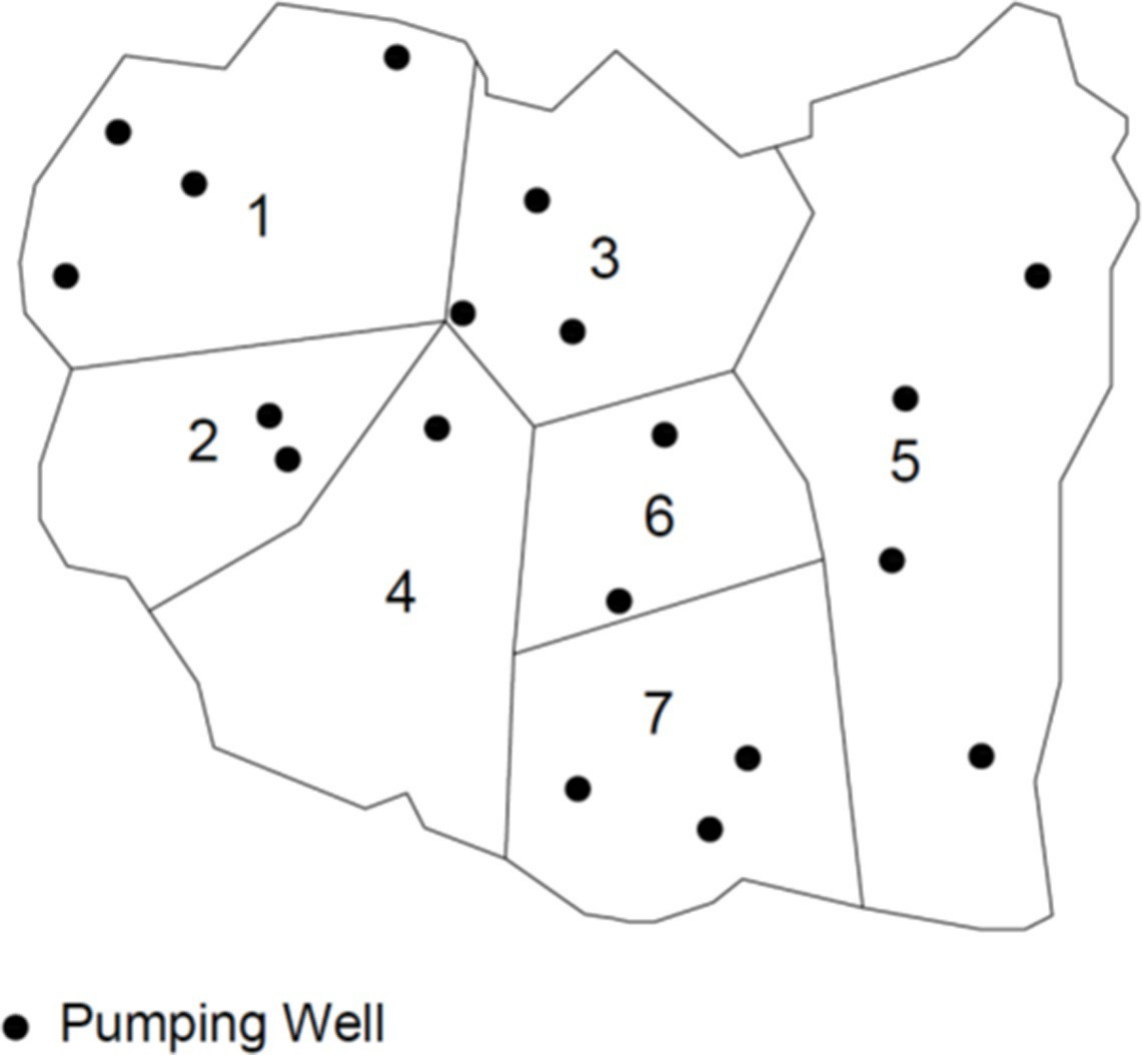
Large-scale test case hydrologic zones and pumping wells.

To build the large-scale test case, the finite-element mesh is taken from a groundwater model constructed for an aquifer in the Oristano plain in west-central Sardinia, Italy [48] and synthetic parameter zones and boundary conditions are applied to the model. The test case aquifer is 11*km x* 9*km* and the finite-element mesh has 29,197 nodes, 57,888 elements, seven parameter zones, 20 pumping wells, and is surrounded by constant head boundaries (Fig 11). This is a highly discretized, realistic groundwater model.

**Fig 12:**
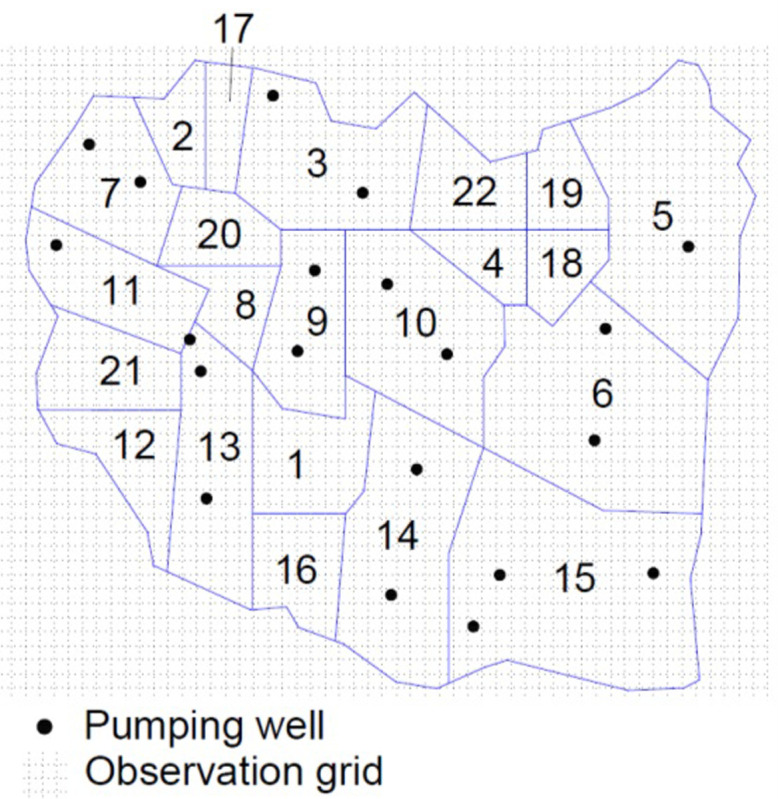
Large-scale test case experimental design setup.

This is the same test case used by Ushijima and Yeh [10] to test their robust experimental design algorithm. Following their methodology, a reduced model is constructed that has a size (*n*_*p*_) of 103, and a dimension reduction of two orders of magnitude. After constructing the reduced model, the aquifer is divided into 22 observation/pumping zones and for practicality, overlay a course observation grid that has a discretization of 400*m* (Fig 12).

**Fig 13:**
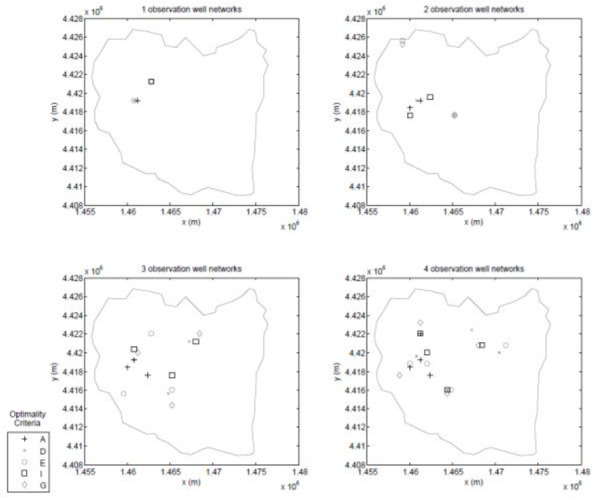
One-, two-, three-, and four-well designs.

**Fig 14:**
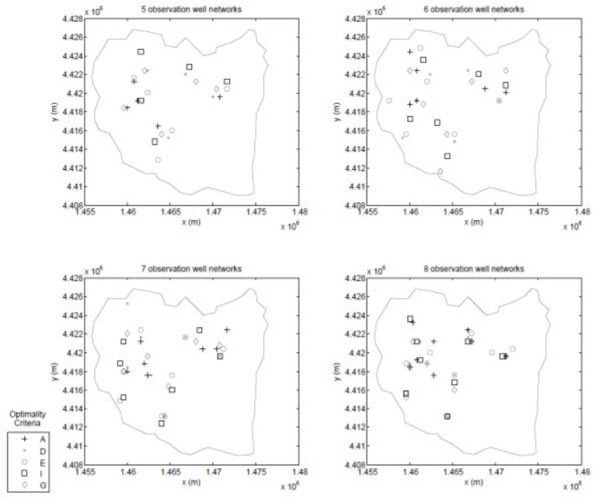
Five-, six-, seven-, and eight-well designs.

**Fig 15:**
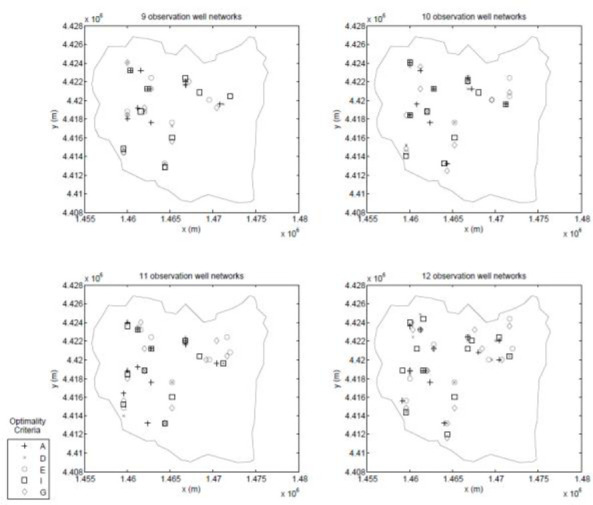
Nine-, ten-, eleven-, and twelve-well designs.

A feasible observation location is defined as a location that falls within an observation zone (i.e. an observation/pumping zone without a pumping well) and on a node of the observation grid. After imposing these constraints, it is found that there are 531 feasible observation locations. Then, seeking information on the hydraulic conductivity with the experimental design, a robust experimental design following the methodology of Ushijima and Yeh [11] is constructed. Previous research by Sun and Yeh [49] demonstrated that for a robust experimental design for hydraulic conductivity, it is sufficient to consider only the upper and lower bounds of the hydraulic conductivity, so upper and lower bounds of 0.1*m*/*day* and 20*m*/*day* are assumed for the hydraulic conductivity in all parameter zones and consider all possible combinations of zonal hydraulic conductivity. As with the first test case, both a GA and a PSO are used to search for the solution to the experimental design problem. In this case, one- through 12-well designs are developed for each of the optimality criterion listed Table 1. These two sets of designs are combined to form a best set whose locations are shown in Figs 13–15.

Again, as with the small scale test case, generalized statements are hard to make since these results are tied to this specific experimental design, but some interesting results are seen. The general trends observed in the small-scale test case again appear in this realistically-scaled test case; for example, with regards to the spatial distribution of the different designs the A-designs tend to group the observation wells close to the area of the aquifer that has the highest concentration of pumping wells, while the other criteria tend to spread the observation wells throughout the aquifer.

**Fig 16:**
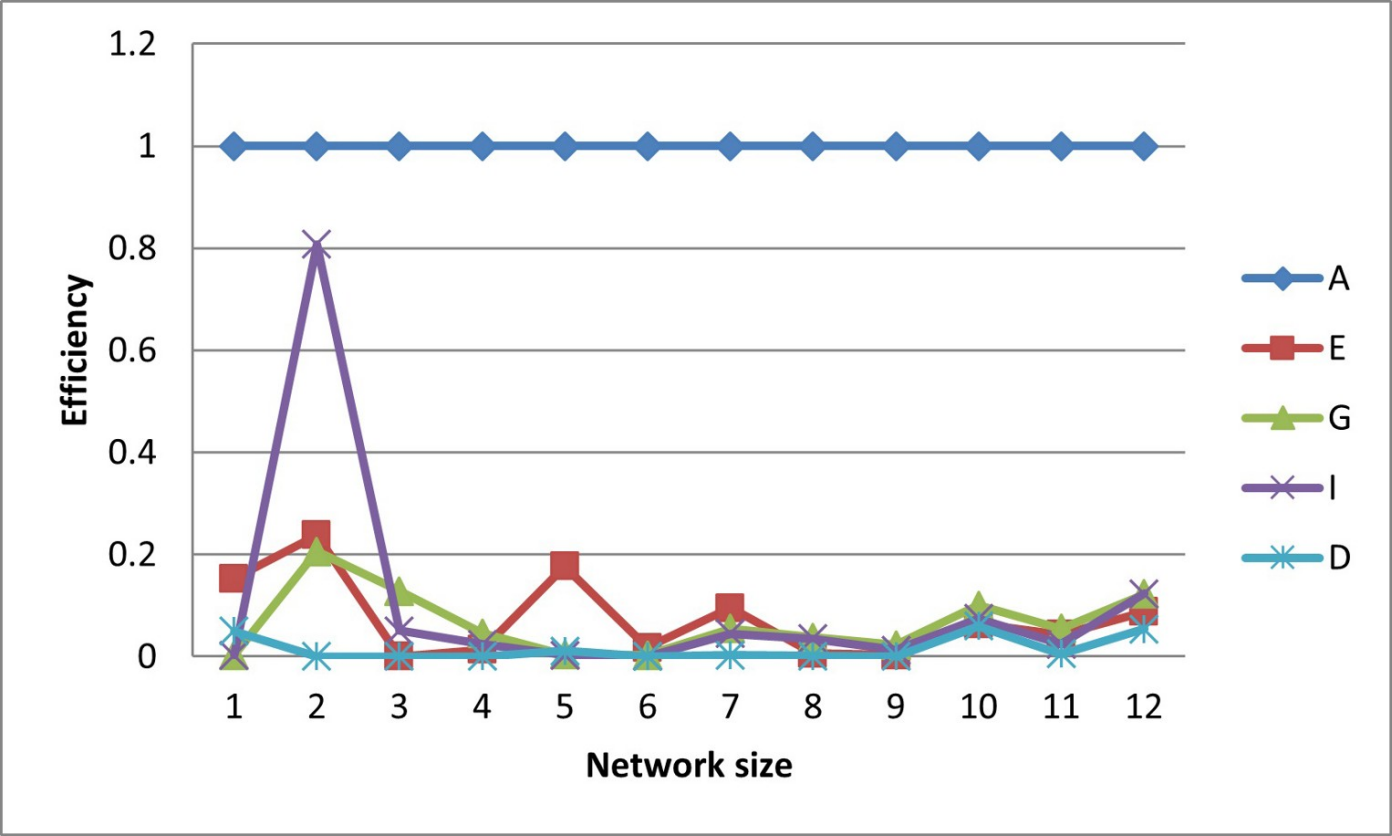
A-efficiencies of various optimal designs for the second case study.

**Fig 17:**
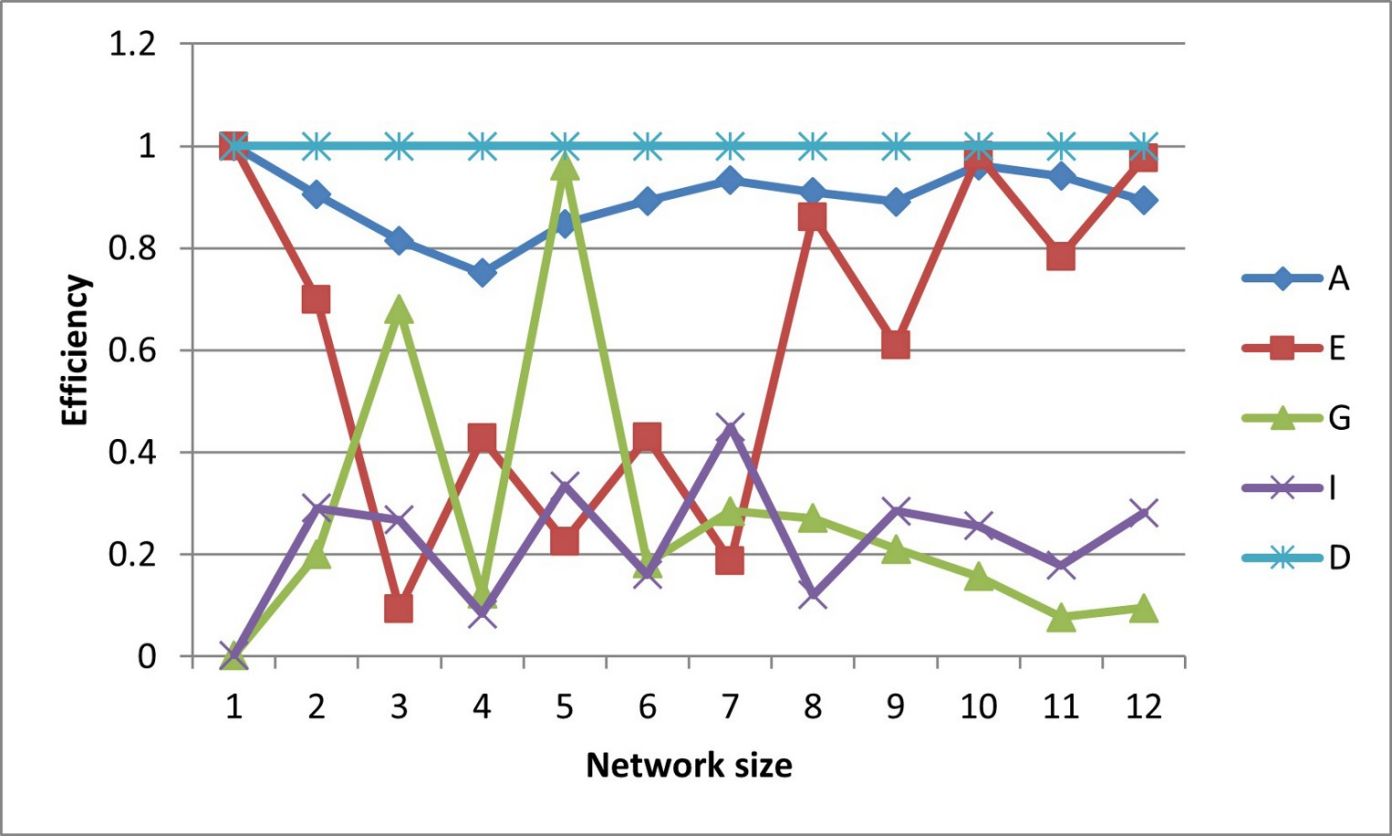
D-efficiencies of various optimal designs for the second case study.

**Fig 18:**
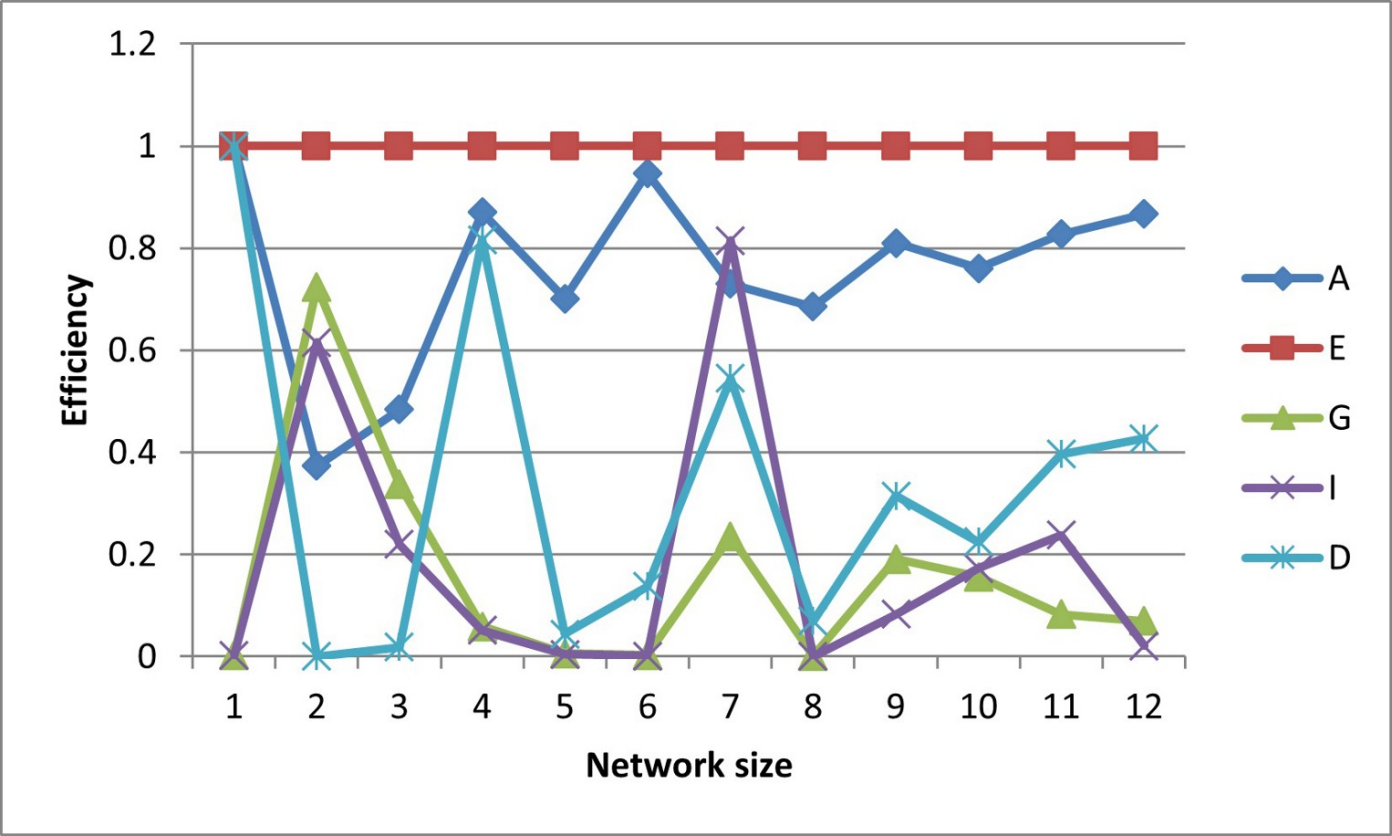
E-efficiencies of various optimal designs for the second case study.

**Fig 19:**
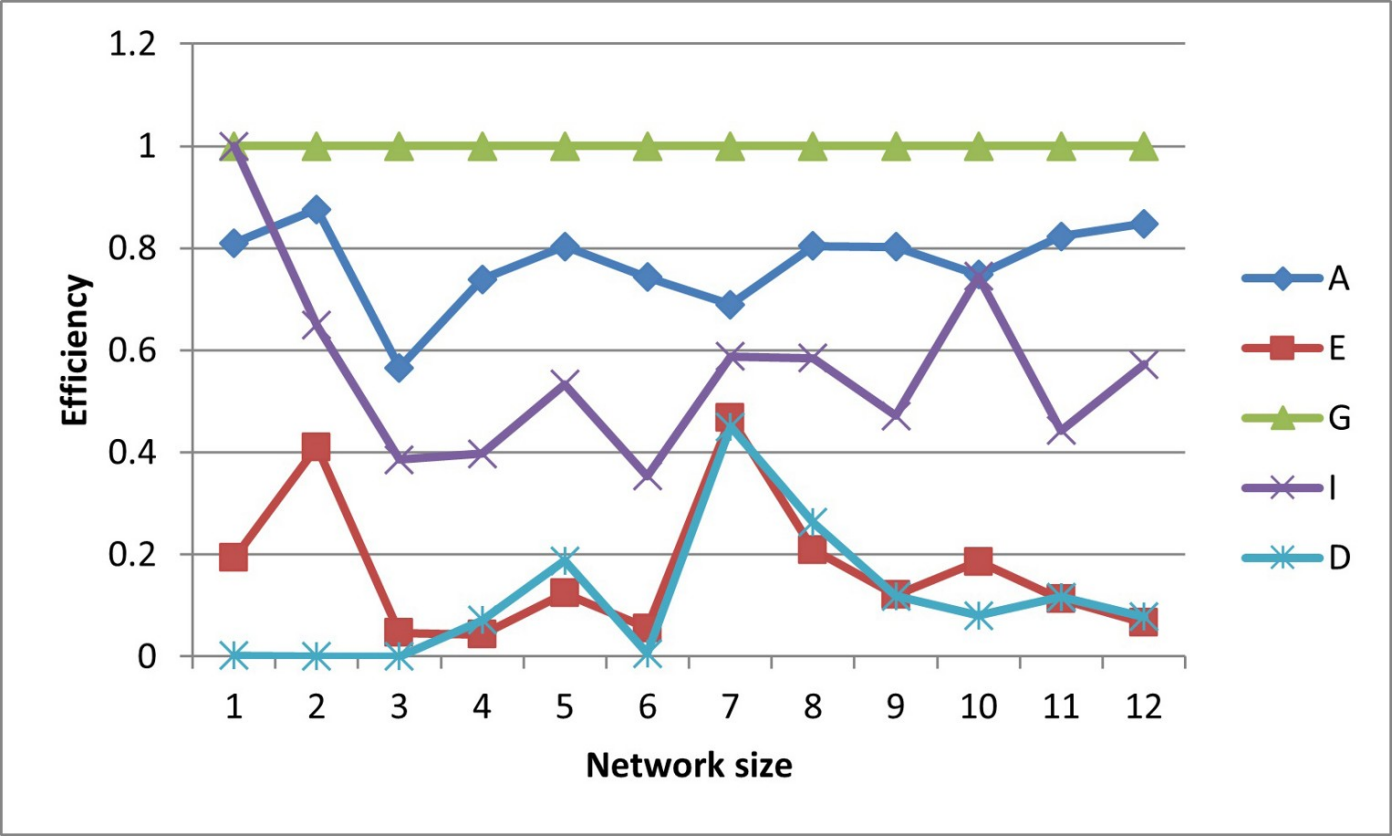
G-efficiencies of various optimal designs for the second case study.

**Fig 20:**
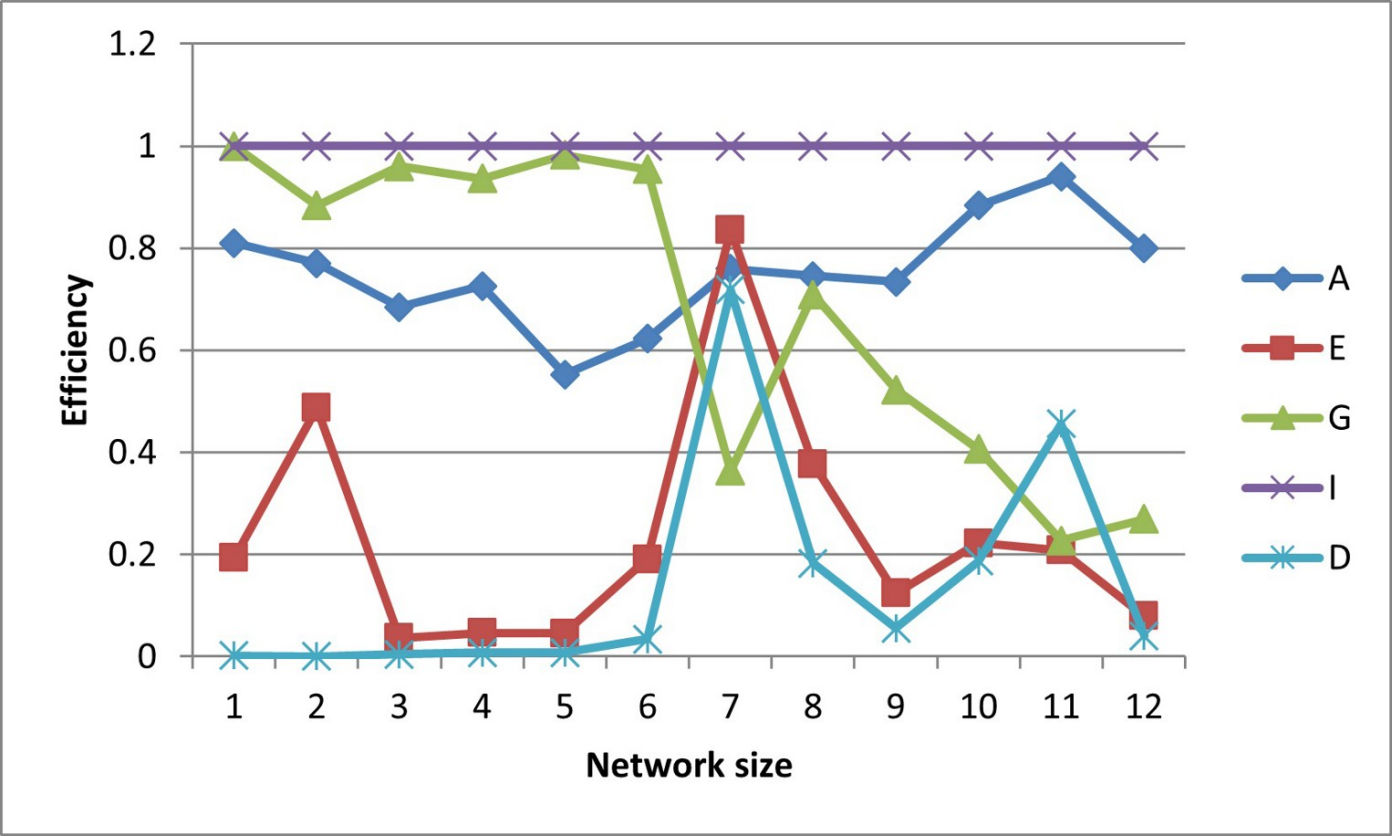
I-efficiencies of various optimal designs for the second case study.

In terms of efficiencies (graphed in Figs 16–20), the designs for the realistically-scaled test case also behave similarly to the designs for the small test case. All the designs are fairly A-optimal but the A-designs are not non-criterion-optimal. Though less obvious than in the small-scale test case, the E- and D-designs are relatively optimal with respect to each other. In general, the prediction-designs (G- and I-designs) are more optimal with respect to each other than with respect to the parameter-designs (A-, D-, and E-designs), while similarly the parameter-designs are more optimal with respect to each other than with respect to the prediction-designs.

One interesting feature that, while present, is not as noticeable in the small-scale results is that for the smaller designs, there are many instances that the D-, G-, and I-efficiencies are all virtually equal to zero, indicating that these designs captured minimal information about these criteria. This behavior makes sense, as it could be difficult for a small number of observation wells to obtain sufficient information to characterize a large aquifer.

While analyzing the results, it is again observed that the PSO tends to outperform (with respect to objective function score) the GA for the smaller designs (i.e. those with smaller observation well networks) while the GA outperforms the PSO for the larger designs (see S1 File in S1 File, S1 Fig in S1 File., and S1 Table in S1 File for additional discussion). In addition, both the GA and PSO again fail to search-out all the best-possible locations. For the GA, the five-well I-design is outperformed (with respect to I-optimality) by the five-well G-design, and the two- and seven-well E-designs are outperformed by the G- and D-designs of the same size, respectively. Similarly, the PSO converges to a three-well G-design that has an I-efficiency greater than 100%. For both algorithms and both test cases these algorithmic issues may be alleviated by either tuning the algorithm parameters. As noted before, as heuristic algorithms are not the focus of this study, this tuning was not carried out.

## 5. Discussion and conclusions

The A-, E-, D-, G-, and I-optimality criteria are analyzed and their applicability is demonstrated to a groundwater experimental design problem. The analysis compares the properties of the different optimality criteria, including what information each seeks, as well as challenges that could be faced in implementing the criteria in an experimental design problem. The largest challenge is the numerical instability encountered when computing the objective function for some of the criteria, specifically D-, G-, and I-optimality. To overcome this difficulty, techniques are suggested that could be used to stabilize the computation of these objective functions. It is shown how heuristic searches coupled with POD-reduced models could be used to find various types of groundwater optimal experimental designs and how the concept of optimal efficiency can be employed usefully to compare the relative performance of competing designs before implementation. Although not studied in this paper, another immediate application of the concept of design efficiency is to ascertain robustness properties of a design to model assumptions. This is of practical interest because the postulated model is often specified with some uncertainty and so it is desirable to implement a design that retains high efficiencies under slightly changed models, which includes misspecifications in the nominal parameters in the model. The results are specific to the test cases presented but some general conclusions can be drawn. One observation is that the optimal designs for the models are not necessarily efficient under another criterion. This is especially true when the optimality criteria are seeking the different types of information, i.e. information about model parameters versus model predictions. For the test cases, among the criteria seeking information about model parameters, it is observed that the D- and E-designs are fairly optimal with respect to each other (i.e. had high efficiencies) and exhibit fairly high A-efficiencies; however the A-designs produce very low D- and E-efficiencies. These results make sense in light of the fact that A-designs seek the maximum amount of information, while D- and E-designs seek the highest quality information (uncorrelated or unique data, respectively). The maximum amount of information may be highly correlated or not unique, leading to low D- or E-efficiencies; however, high quality information should contain a large amount of information, leading to a high A-efficiency. The test case results show that the optimal designs for model prediction (G and I) do not have high model parameter efficiencies. This might be expected, as different model parameter values could produce similar predictions. However, it was surprising to see that optimal designs for model parameters (A, D, or E) did not necessarily produce high model prediction efficiencies, as one might expect a model with parameter values close to the true values would produce good model predictions.

In this study, a reduced model was developed using POD and coupled with GA and PSO to solve the combinatorial optimization problem for experimental design. One downside of heuristic searches is that there is no guarantee that the distribution of the location wells found by the algorithm is globally optimal; rather, it shows just that it is more optimal than all visited locations. This leads to the risk that the search would converge to a sub-optimal solution, particularly when the solution space is difficult to search. In the context of experimental design, the solution spaces could have large areas whose score is very close to the computer’s round-off error threshold (particularly with the D- and E-optimality criteria), leading to difficulties for the algorithms in determining search directions. In addition, these types of solution spaces tend to have very spiky optimal regions, making it easy for the algorithm to bypass the global optimum. An optimality criterion, such as G-optimality, may have a very discontinuous solution space that could lead to the algorithm getting stuck on one side or the other of a “cliff”. In terms of heuristic searches, the A-optimality criterion presents the most searchable solution space, as its formulation (a summation) lends itself to a relatively smooth and well-behaved solution space. In this study, it is observed that there is evidence that both the GA and PSO got stuck in sub-optimal solutions, as many GA- and PSO-designs had non-criterion efficiencies greater than 100%–something that would be impossible had the searches converged to the global optima. While this is of course undesirable, many times the sub-optimal solution appeared to be a good estimate of the best-possible solution. For example, Fig 9 shows the I-optimality of the five-well G-design is 102%. This inspires confidence that in general, a GA or a PSO, will find at least good approximate solutions to the experimental design problems. A second and perhaps more significant drawback of heuristic searches is the large number of model calls required to achieve convergence of solution. POD model reduction effectively combats this challenge by allowing the user to control the tradeoff of model accuracy vs. model speed. Some increase in speed will always be achieved but depending on how much uncertainty in result the user is willing to accept, more speed may be acquired at the cost of more uncertainty.

The self-created computer code used in this study is provided in the below github repository and it was used to generate all Figs to gain further insights on the choice of the design for the design problem. The Figs showing the design points of the various optimal designs are helpful because they tell us about the distribution of the design points and how their proximity aquifer features (pumping wells, hydrologic zones, etc.) varies within the experimental region. The user may decide that the A and D-optimality are equally appealing but the geography and hydrology properties of the experimental region may make it impracticable or expensive to take observations at several design points required by the A-optimal design and not by the D-optimal design. Likewise, the Figs showing how the efficiencies of an optimal design vary under a change of criteria provide meaningful information to the user for choosing a robust criterion for implementation. For example, consider results from our first case study. Fig 6 shows none of the other optimal designs has acceptable A-efficiencies regardless of the network size; among the E, G, I and D-optimality criteria, the maximum A-efficiency attained was by the D-optimal designs across different network sizes even though the A-efficiency attained is only 60%. In contrast, Fig 7 suggests that for all practical purposes, E-optimal designs are almost as efficient as the D-optimal designs for making inference on the model parameters across different network sizes. This is followed by A-optimal designs. The other two optimal designs, I and G-optimal designs performed best when the network size is 6 and both attained a D-efficiency of about 55%; for the other network sizes, Fig 7 shows their D-efficiencies are not more at most 40%. The same interpretation and implications can also similarly be made for the second case study. Consequently, the Figs presented can assist the user in selecting an optimality criterion and an optimal design using case studies before they are implemented in practice.

While our results and interpretations apply to the specific case under consideration and do not generalize, our codes can be amended to facilitate similar and meaningful evaluations for a real application. The only modification that would be required is to replace the test case reduced model with a reduced model of a real world case which may be constructed following the techniques outlined in this paper. With very little modification, our codes are designed to be modular with easy swapping out of optimization algorithm and the reduced model. By swapping or optimizing the algorithm, the user may achieve quicker and/or more optimal results (since metaheuristic algorithms do not guarantee optimal solutions). More importantly, the reduced model of the test cases may be swapped for a reduced model of a real world case so that the techniques applied in this study may be applied in a real-world situation.

In conclusion it is emphasized that it is imperative to have a clear understanding of what information is being sought at the start of any wells allocation study so that an appropriate optimality criterion can be chosen. The computation complexities faced by an experimental design problem must also be understood and pro-active steps be taken. If heuristic searches are used to overcome some of the computational complexities, their own computational complexities also must be dealt with. In addition, the choice of an algorithm must be made wisely, as not all searches perform equally well. Despite these challenges heuristic searches, particularly when coupled with reduced models, may be the only option to solve realistically-scaled experimental design problems. In the future, this study could be expanded to include other heuristic searches such as randomized PSOs [37] or Cuckoo searches [50]. Alternate design methods also could be explored, such as hybrid searches or methods that identify critical design points within a groundwater model. Examples include the Greedy Algorithm proposed by Boyce et al. [51] and the discrete empirical interpolation method (DEIM) [52], or extension using a Bayesian formulation [53]. A recent example of a hybrid algorithm is Shi et al. [54], who combined an advanced version of PSO called quantum PSO and Random Forest to predict disease progression of patients with idiopathic pulmonary fibrosis.

## Supporting information

S1 File (DOCX)
