# Supplementary material for: Constructing robust and efficient experimental designs in groundwater modeling using a Galerkin method, proper orthogonal decomposition, and metaheuristic algorithms
Source: PLoS One. 2021 Aug 5;16(8):e0254620. doi: 10.1371/journal.pone.0254620 (PMC8341624; doi:10.1371/journal.pone.0254620)
Supplement: S1 File — (DOCX) [file pone.0254620.s001.docx]

**S1 File. Test Results from the GA and PSO Algorithms**

We compared performance of the two algorithms using simulation by replicating the results 10 times. The termination rule for each algorithm was that the algorithm converged as described in the schematics below in not less than ten generations for GA and 10 position and velocity updates for PSO (S1 Fig.). Following the termination of an algorithm, the algorithm was reinitialized until ten replicates each of the GA and PSO results had been collected. The aim of the simulation study was to test the null hypothesis that the efficiencies of designs found by GA and PSO averaged over the five (A, E, I, D and G-optimality) criteria are equal. The alternative hypothesis is that their averages are unequal at the 0.05 significance level.

The choice of the set up for the simulation was motivated mainly by the CPU time required because millions of model calls were required to perform the test. For example, on an Intel Core 2.40 GHz i5 CPU, O(1,000,000) reduced-model calls require 7.5 hours to complete while O(1,000,000) reduced-model calls only require 0.75 hours to complete. More specifically, combined with the overhead for each algorithm, each PSO (S1) replicate took about a day to complete while each GA (S2) replicate took about 4 days to complete. It is also for this reason that the performance test was limited to the twelve-well because we expect discrepancies in performance will be magnified with larger designs and smaller designs for smaller number of wells is likely to show no discrepancy in the test result. Table S1 reports the averaged efficiencies of designs found from the two algorithms

S1 Table. Averaged design efficiencies from the GA and PSO with 10 replicates for the twelve-well designs.

| Replicate  Algorithm | 1 | 2 | 3 | 4 | 5 | 6 | 7 | 8 | 9 | 10 |
| --- | --- | --- | --- | --- | --- | --- | --- | --- | --- | --- |
| GA | 36% | 34% | 33% | 38% | 35% | 39% | 35% | 35% | 37% | 35% |
| PSO | 32% | 44% | 47% | 43% | 41% | 39% | 35% | 40% | 31% | 40% |

We first employed graphical tools to evaluate whether the averaged efficiencies were normally distributed and they were clearly not. The rank-sum test was then used to test the null hypothesis and the p-value was 0.07. The PSO replicates tended to have higher efficiencies than those from GA, but the null hypothesis that the GA and PSO performed equally well cannot be rejected. We note that the GA replicates required O(1,000,000) reduced model calls to terminate, while the PSO replicates required only O(100,000) model calls to terminate, implying that default tuned PSO was more suited for this particular heuristic search than the default tuned GA.

The test from the non-parametric test does not suggest the average design efficiencies from the two algorithms are statistically significant. Even if it slightly did, we are not overtly concerned for several reasons. First, it is not surprising because there is the No Free Lunch Theorem that asserts that no one heuristic algorithm can perform better than all other heuristic algorithms for all situations. Second, there are many ways to improve the performance of a heuristic algorithm by tuning the parameters in the algorithm. Our goal was never to find the best algorithm for finding highly efficient designs for water research as there will be too many metaheuristic algorithms to compare and so we were using these two popular heuristic algorithms in water research with default values for the tuning parameters. Further, there are many enhanced versions of GA and PSO, including choices for the tuning parameters with mathematical justifications; two recent work in this direction are Tong, et al. (S3) and Choi et al. (S4). The optimization of either GA or PSO is beyond the scope of this research and seems tangential to our main purpose here, which is to promote the use of design efficiencies in water research work.

**S1 References:**

S1. Pedersen MEH. SwarmOps [Internet]. Hundested, Denmark: Hvass Labs; 2008. p. 1–76. Available from: https://github.com/Hvass-Labs/swarmops-other

S2. Wall M. GAlib: A C++ Library of Genetic Algorithm Components [Internet]. galib247. Cambridge: Massachusetts Institute of Technology (MIT); 1995. p. 1–104. Available from: http://lancet.mit.edu/ga/dist/

S3. Tong XT, Choi KP, Lai TL, Wong WK. Stability bounds and almost sure convergence of improved particle swarm optimization methods. Res Math Sci [Internet]. 2021 Jun 5;8(2):30. Available from: https://link.springer.com/10.1007/s40687-020-00241-4

S4. Choi KP, Lai TL, Tong XT, Wong WK. A Statistical Approach to Adaptive Parameter Tuning in Nature-Inspired Optimization and Optimal Sequential Design of Dose-Finding Trials [Internet]. Stanford, California; 2020. Available from: https://statistics.stanford.edu/research/statistical-approach-adaptive-parameter-tuning-nature-inspired-optimization-and-optimal

**S1 Fig. Convergence schematics.**

**
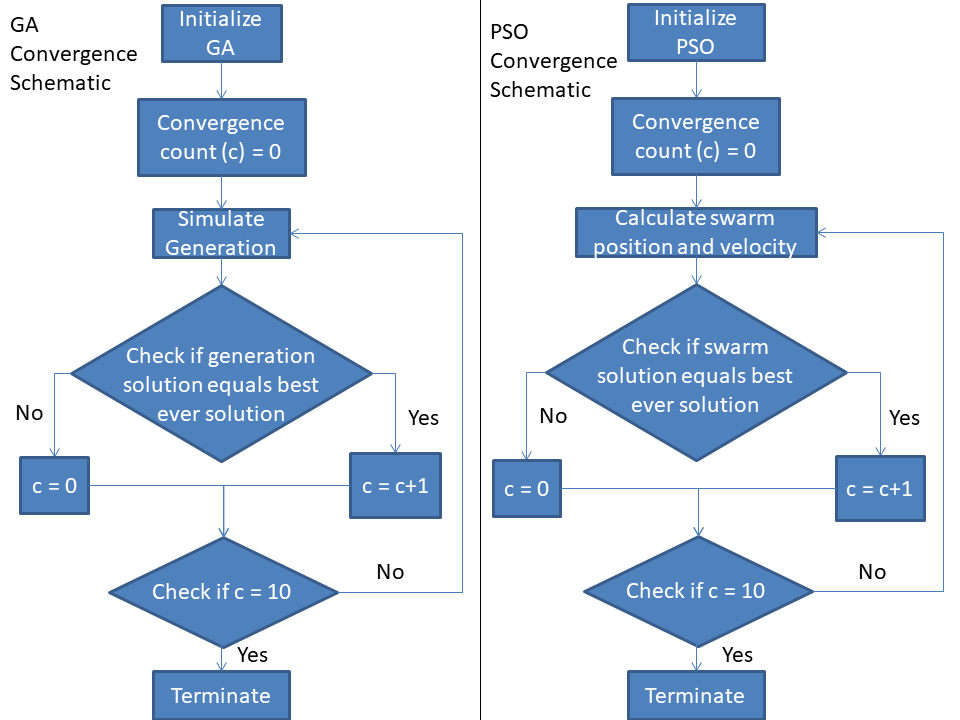
**
